# URDME: a modular framework for stochastic simulation of reaction-transport processes in complex geometries

**DOI:** 10.1186/1752-0509-6-76

**Published:** 2012-06-22

**Authors:** Brian Drawert, Stefan Engblom, Andreas Hellander

**Affiliations:** 1 Department of Computer Science, University of California, Santa Barbara, California 93106, USA; 2 Department of Information Technology, Uppsala University, Uppsala 75105, Box 337, Sweden; 3 UPMARC, Uppsala Programming for Multicore Architectures Research Center, Uppsala, Sweden

## Abstract

**Background:**

Experiments *in silico* using stochastic reaction-diffusion models have emerged as an important tool in molecular systems biology. Designing computational software for such applications poses several challenges. Firstly, realistic lattice-based modeling for biological applications requires a consistent way of handling complex geometries, including curved inner- and outer boundaries. Secondly, spatiotemporal stochastic simulations are computationally expensive due to the fast time scales of individual reaction- and diffusion events when compared to the biological phenomena of actual interest. We therefore argue that simulation software needs to be both computationally efficient, employing sophisticated algorithms, yet in the same time flexible in order to meet present and future needs of increasingly complex biological modeling.

**Results:**

We have developed URDME, a flexible software framework for general stochastic reaction-transport modeling and simulation. URDME uses **U**nstructured triangular and tetrahedral meshes to resolve general geometries, and relies on the **R**eaction-**D**iffusion **M**aster **E**quation formalism to model the processes under study. An interface to a mature geometry and mesh handling external software (Comsol Multiphysics) provides for a stable and interactive environment for model construction. The core simulation routines are logically separated from the model building interface and written in a low-level language for computational efficiency. The connection to the geometry handling software is realized via a Matlab interface which facilitates script computing, data management, and post-processing. For practitioners, the software therefore behaves much as an interactive Matlab toolbox. At the same time, it is possible to modify and extend URDME with newly developed simulation routines. Since the overall design effectively hides the complexity of managing the geometry and meshes, this means that newly developed methods may be tested in a realistic setting already at an early stage of development.

**Conclusions:**

In this paper we demonstrate, in a series of examples with high relevance to the molecular systems biology community, that the proposed software framework is a useful tool for both practitioners and developers of spatial stochastic simulation algorithms. Through the combined efforts of algorithm development and improved modeling accuracy, increasingly complex biological models become feasible to study through computational methods. URDME is freely available at http://www.urdme.org.

## Background

Stochastic simulation of reaction kinetics has emerged as an important computational tool in molecular systems biology. In cases for which mean-field analysis has been shown to be insufficient, stochastic models provide a more accurate, yet computationally tractable alternative [1-3]. For example, a frequently studied topic is the mechanisms for robustness of gene regulatory networks relative to intrinsic and extrinsic noise [4-6]. In a stochastic mesoscopic model the time evolution of the number of molecules of each species is described by a continuous-time discrete-state Markov process. Realizations of this process can be generated using techniques such as the Stochastic Simulation Algorithm (SSA) [7].

If the system can be assumed to be spatially homogeneous, or well-stirred, simulations are simplified considerably compared to a spatially varying setting. However, there are many phenomena inside the living cell for which spatial effects play an important role [8,9]. In such cases, a mesoscopic spatial model can be formulated by first discretizing the computational domain into subvolumes, or voxels. Molecular transport processes are then modeled as combined decay- and creation events that take a molecule from one voxel to an adjacent one [10,11]. For appropriate discretizations [12,13], the assumption of spatial homogeneity holds approximately within each voxel, where reactions can be simulated as in the well-stirred case. The governing equation for the probability density function is called the Reaction Diffusion Master Equation (RDME) and methods to generate realizations in this framework have been used previously to study reaction-diffusion systems in the context of molecular cell biology [8,14-16].

Modern experimental techniques can provide information not only on the total copy numbers but also on the spatial localization of individual molecules [17,18]. As such techniques are further developed and spatial models can be calibrated to biological data, methods and software for flexible and efficient simulation of spatial stochastic models will likely continue to grow in importance. As a coarse-grained alternative to detailed microscopic models based on Smoluchowski reaction dynamics [19,20], or other similar microscale simulators such as MCell [21], simulations in the RDME framework are orders of magnitude faster than microscopic alternatives [22].

For most applications, a large number of sample realizations need to be generated to allow for a useful statistical analysis. Exploring parameter regimes or estimating responses to different stimuli adds to the complexity so that the generation of tens of thousands of independent realizations is not uncommon. Computational efficiency is therefore an important concern and has motivated research in many types of approximate or optimized methods (see for example [23-27]).

Despite advances in the development of approximate methods, spatial stochastic simulation in realistic geometries is still challenging. One of the main reasons is the complexity involved in handling the 3D geometry and the associated mesh. The purpose with this paper is to introduce URDME, a modular software framework for spatial stochastic simulation. The goal of URDME is twofold: firstly, it provides applied users with a powerful and user-friendly modeling environment that supports realistic geometries. Secondly, URDME facilitates the development of new computational methods by taking care of the technical details concerning the geometry, the mesh generation, and the assembly of local rate constants. By providing a well-defined interface to the modeling environment, new algorithms can be incorporated into the URDME framework as plug-in solvers. We anticipate that this modular structure will facilitate the development and dissemination of advanced simulation methodologies to real-world molecular biology applications.

URDME differs from other public domain software for mesoscopic simulations such as MesoRD [28] or SmartCell [29], in that it uses unstructured tetrahedral meshes to discretize the domain, offering a much greater geometrical flexibility and better resolution of curved surfaces compared to Cartesian meshes. URDME shares its utilization of tetrahedral meshes with another reaction-diffusion simulation software, STEPS [22], which we will discuss later in the paper. One of the defining features of URDME is that it is structured to be highly modular in order to be useful as a platform for developers of the associated computational tools. This design also allows for flexible work-flows for result generation. When used interactively, URDME’s Matlab interface provides for convenient model construction and evaluation. Since the solvers are automatically compiled into optimized stand-alone executables, URDME can also be used to define batch jobs using the very same Matlab interface. In this way, URDME is a convenient platform both in the initial modeling phase as well as when performing high-performance and/or high-throughput computational analysis.

## Implementation

In this section we describe how the URDME framework is structured, how it is used to simulate a model, and how to interface with it to add new simulation algorithms. For more details we refer to the software manual [30] included in the software distribution (Additional file 1).

### Overview

**Figure 1:**
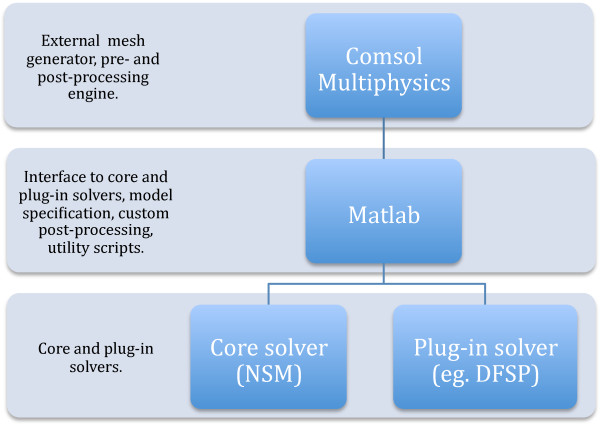
**The URDME framework consists of three loosely coupled layers.** Solvers reside at the bottom level and are most often written in a compiled language like ANSI-C. The middle layer provides for interfaces between the solvers and the top-level mesh-generation infrastructure. Both the top- and the bottom-layer may be replaced by other software as long as the middle level is extended appropriately.

The URDME framework consists of three logical layers connected by well-defined interfaces (see Figure 1). At the top level, a third-party software for mesh-generation is used to define the geometry and to generate the mesh. Currently, URDME interfaces with Comsol Multiphysics 3.5a for this functionality. The middle layer routines in Matlab serve as an interactive environment for model construction, and connects the geometry and mesh-handling facilities of Comsol with the core simulation algorithms (bottom layer).

With this modular structure, the top level can be replaced by other mesh generation software such as for example Gmsh [31], provided that the appropriate interface routines are added to the middle level interface. Relying on Comsol Multiphysics for the geometry definition and mesh-generation provides for a convenient interactive environment for the model construction, allowing advanced models to be formulated quite easily.

The default core solver at the bottom level is an optimized implementation of the Next Subvolume Method (NSM) [8]. Since the solver layer is kept separate from the model building interface, new solvers can easily be added to URDME while taking advantage of all of the infrastructure related to model management and post-processing. The data passed to the solvers is well-defined and documented (see [30] for more information). It is our goal for URDME to grow through the contribution of solvers from the community. One such solver has already been contributed and distributed in this way: the diffusive finite state projection (DFSP) algorithm [32]. Additionally, the URDME framework has been utilized in the development of new algorithms [27,33,34] and a master equation formulation of active transport on microtubules [35].

### Using URDME for model development and simulation

**Figure 2:**
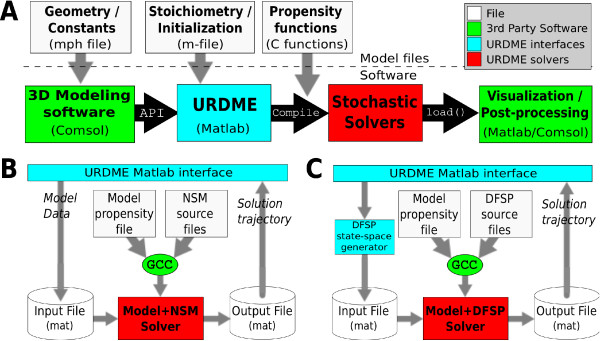
**Summary of the URDME software infrastructure.** (**A**) URDME flow diagram for the complete simulation process. (**B**) Process flow diagram for the stochastic simulation step of (A) using the NSM solver. (**C**) DFSP solver flow diagram, an alternative to (B) for the stochastic simulation step.

The process of analyzing a reaction-diffusion model with URDME begins with the creation of a Comsol model file that defines the geometry of the domain, including (optionally) the subdomains where specific *localized* reactions are to be defined (e.g. membrane, cytosol, and nucleus). At this stage, the biochemical species and their associated diffusion rates are also defined. Once the model is set up, the mesh generation facilities of Comsol are used to create a tetrahedral discretization of the domain. Next, this information is exported to Matlab via an API connection as illustrated in Figure 2A (top). The interface routines of URDME are then used to assemble the data structures needed by the core simulation routines. This whole process is summarized in Figure 2A (bottom).

Apart from defining the geometry, the user also needs to create two additional program files to be used by URDME. The first is a Matlab function (referred to as the *model file*), that defines the data related to the actual simulation. This includes the initial distribution of molecules, the stoichiometric matrix defining the topology of the reaction network, a certain dependency graph for events in the model, and the simulation interval (for a detailed list, see [30]). This model file can also be used to define custom configurations for the model, including restricting a species to a specific subdomain, adding modified transport terms, and evaluating expressions over the geometry such that this information can be passed on to the core solver. In this way, URDME supports custom modeling that would be very hard to achieve with a less flexible software architecture. This, we argue, is one of the defining and unique features of the URDME framework.

The second program file a user must create is a templated C-program file that defines the propensity functions for the chemical reactions of the model. This file defines one function for each chemical reaction in the system and are called by the core solver routines to calculate the propensity for each reaction in each voxel. The propensity function template requires the output to depend only on the system state at the current time, but is unique to a voxel and allows for additional data to be passed on to the function. The propensity function file is later automatically compiled and linked with the core solver, resulting in a highly efficient solution procedure.

Once the model data structure has been exported to Matlab and the model and propensity functions have been defined, the next step is to let URDME execute a simulation of the model. From the users’ perspective, simulation now only requires to invoke the urdme function in Matlab with the proper arguments,

>> model = urdme(model,@model_file, {’Propensities’,’propensity_file’});

The arguments passed are the Comsol data structure, the model function, the propensity functions, and various optional arguments. URDME now invokes GCC to compile the propensity function file with the specified solver (defaulting to NSM) to create a dedicated executable for the model. This executable is then invoked using the model and geometry data structure as inputs. Note that compilation and execution of the low-level components of the system is fully automatic, and requires no additional action from the user. Following a successful execution of the core solver the urdme function returns a modified model data structure with a single stochastic solution trajectory attached to it.

Since the layers of URDME are decoupled, it is also possible to execute the solvers in non-interactive batch mode to allow for more flexible result generation and distribution of computations on a multicore platform. For example, to conduct the simulation in background mode and write the resulting trajectory to the file ‘output.mat’ one simply invokes urdme with a few additional arguments,

>> model = urdme(model,@model_file, {’Propensities’,’propensity_file’, ... ’Mode’,’bg’,’Output’,’output.mat’});

Here, control returns to Matlab directly after execution of the solver executable, without waiting for it to complete.

Visualization and post-processing are important components in most simulation software. Once a URDME simulation is complete, users can easily visualize the spatially varying concentration of biochemical species in their model by using Matlab’s interface to the Comsol graphics routines. Examples of this will be presented in the Results section. Similarly, most modeling and simulation projects require custom data analysis once the simulation data has been generated. To facilitate this, URDME supports the creation of post-processing scripts in Matlab using its native high-level scripting language and computational libraries. Examples of complex post-processing routines implemented as Matlab functions and scripts are available as part of the example directories in the URDME software distribution package, and in Additional file 2, Additional file 3 and Additional file 4.

### Structure and implementation of core simulation algorithms

Taken together, the components of URDME that was introduced in the previous section create a flexible and expandable platform. While an applied user need not know any details about how a core solver is implemented, a developer of a new simulation algorithm can use the infrastructure to develop a plug-in solver to URDME. Figure 2C illustrates the structure of the plug-in solver that implements the DFSP algorithm [32]. Note the similarities with the flow diagram of the core NSM solver in Figure 2B. URDME plug-in solvers have three main components: a Makefile, the solver source files, and (optionally) a pre-execution script intended to be invoked by the middle-level scripting interface. The solver Makefile is used for compiling and building the solver automatically from the Matlab interface. The name of this file tells URDME what solver it builds; when urdme is invoked with the option to run a simulation using a specific solver, it will look for a Makefile with the correct naming pattern. This Makefile then compiles the solver along with the propensity functions associated with the model being simulated into a stand-alone binary executable. Hence a different and unique executable is automatically produced for each new combination of model and solver.

The source code of the solver itself can formally consist of any number of files in any language as long as the Makefile can create the final executable called by the middle-level interface. To enable a seamless integration with the URDME Matlab interface, the URDME C API contains library routines to read and parse the data structures generated by the URDME model files. These API routines will parse all data-structures required by the core NSM solver. A plug-in solver that needs additional input will have to make sure that these are parsed correctly as part of the solver main routines. To pass such additional data to the solver, it need only be appended to the ‘model.urdme’ field, either by the Matlab model file, or by a pre-execution script (compare Figure 2C). URDME will then write this data to the solver input file. Such a pre-execution script is an optional component of the solver integration. Simply put, when executing a model, URDME always looks for a Matlab function defined in the file ‘urdme_init_<*solver*>.m’.

All current solvers are written in ANSI-C and use GNU-style Makefiles. The process of integrating a simulation algorithm in the URDME framework is described in more detail in [30] and is also exemplified by the source code for the DFSP plug-in that is included in the URDME distribution.

In conclusion, when all the components of a solver is in place as described above, the only difference to an end-user of URDME is a single additional argument

>> model = urdme(model,@model_file, {’Propensities’,’propensity_file’, ... ’Solver’,’dfsp’});

The use of the URDME framework to implement and analyze the performance of a simulation algorithm will be further exemplified in the Results section.

## Results

In this section we will use three different examples to illustrate how the design of URDME makes the software framework a useful tool to accomplish different simulation tasks.

In the first example we show how an established model from the molecular systems biology literature is simulated in URDME. This example illustrates the powerful nature of the URDME scripting environment in setting up and conducting a parameter sweep.

In the second example we demonstrate how URDME can aid in the development of efficient simulation algorithms by explaining how a novel method, the Diffusive Finite State Projection (DFSP) [32], was integrated into URDME as a plug-in solver.

As a final example we simulate a model of molecular transport in a neuron. Here, the unstructured mesh is a critical feature in order to be able to resolve the complex geometry with a feasible number of voxels. We also show with this example how a model of active, molecular motor driven transport as proposed in [35] can be implemented in URDME to simulate molecular transport in the different parts of the neuron.

### Simulating Min oscillations in *E. Coli*

In *E. Coli*, the Min family of proteins are believed to play a key role in the regulation of symmetric cell division. In a mechanism thought to be self-organized and to function in a manner similar to the formation of Turing-patterns, the MinD protein oscillates from pole to pole with a period close to 40 seconds. Another Min protein, MinC, co-localizes with MinD and acts as a repressor for the formation of the cell division site by destabilizing Ftz polymerization [36]. On average, MinD (and hence MinC) will spend less time near the center of the cell, allowing the division ring to assemble there. Both deterministic and stochastic models of this system have been studied previously in the literature [8,36].

**Figure 3:**
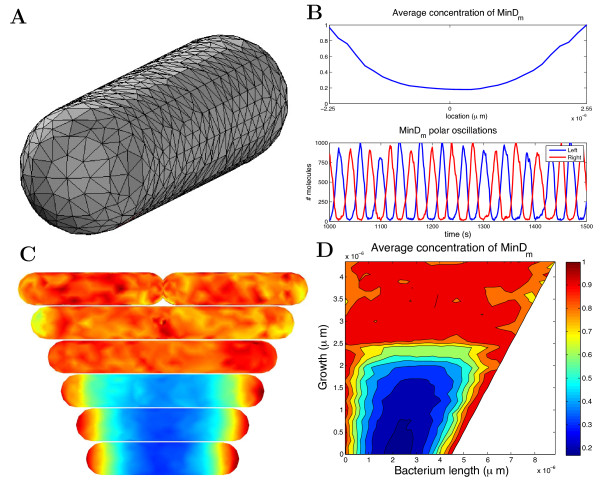
**Simulating Min oscillations in*E. Coli*for varying length of the cell.** (**A**) Geometry and mesh modeling of an *E. Coli* cell. (**B**) Temporal average concentration of MinD protein as a function of position along the long axis of the *E. Coli* cell (top), and the time series plot of the oscillations. (**C**) Six *E. Coli* cells of increasing lengths, as specified in the parameter sweep described in the code block above. The color intensity shows the temporal average concentration of MinD protein along the membrane. (**D**) Parameter sweep shows how the relative concentration of MinD changes as the bacterium grows.

To illustrate how to use URDME to conduct a parameter sweep we will simulate the Min-system for increasing lengths of the bacterium and observe the behavior of the oscillations. The example is representative for how experiments using different sets of parameters can be defined and organized with the current version of URDME. A detailed account for how to create all model files to run simulations of the model from [8] can be found in the software manual [30] in the form of a tutorial. There, the model is run interactively from the Matlab prompt as detailed in the previous sections. In order to conduct the experiment outlined here in the same fashion we would have to manually rebuild the geometry and execute the simulations for the different parameter cases. This would be time-consuming and error prone. Instead, here we exemplify how to automate such a task by using the Matlab scripting environment and the URDME Matlab interface. The code block below shows how the parameter sweep can be specified in a simple script in the Matlab language. The function ‘coli_model’ (Additional file 2) was automatically generated from the Comsol interface using the model of an *E. coli* bacterium shown in Figure 3A. It was then slightly modified by manipulating the original consecutive solid geometry (CSG) description. The geometry of the bacterium is parametrized by creating a copy of the original geometry and then translating it along the x-axis. The union of these two objects is the final geometry and the variable ‘xsep’ specifies the extent of the translation. Note that, as shown in Figure 3C, the bacterium will ultimately split into two separate geometric objects.

Below we show a Matlab script that simulates the Min *E. Coli* model with varying cell length. % Define the parameter space Nval = 30; xsep = linspace(0,4.5e-6,Nval+1); xsep(end) = []; % (avoid creating two % distinct bacteria) save results/info.mat xsep for i = 1:Nval 

 % Generate the E. coli cell by

 % merging two cells with separation

 % xsep(i) along the positive x-axis

 fem = coli_model(xsep(i));

 % run an instance of URDME in

 % background mode

 fem = urdme(fem,@huang,

 {’Propensities’,’huang’, ...

 ’Mode’,’bg’, ...

 ’Outfile’,sprintf

 (’results/out%d.mat’,i)});

 % save input separately for later use

 save(sprintf(’results/in%d.mat’,i)

 ,’fem’);

end

The results of the parameter sweep is summarized in Figure 3. Figure 3A shows the geometry of a model of an *E. Coli* bacterium with length 4.5 *μm*and radius 0.5*μm*discretized with a tetrahedral mesh. Figure 3B shows the temporal average of membrane bound MinD obtained in a simulation of the model from [36] with URDME, as well as a time series of pole-to-pole oscillations of the membrane bound fraction of MinD. As can be seen, the model predicts a minimum of MinD near the center of the cell. Figure 3C shows a visualization of the *E. Coli* bacterium at six different lengths, including the temporal average of the relative concentrations of the MinD protein. Figure 3D shows the stability of oscillations when increasing the ‘xsep’ parameter.

For values of the parameter ‘xsep’ less than about 2*μ*m, coherent oscillations are observed and the MinD protein is concentrated at the poles of the bacterium. For larger values, the oscillations cease and MinD is distributed evenly in the cell. Hence, in order to maintain oscillations also for longer cells, the model needs to be modified in some way. For example, the total copy number of MinD is currently kept constant as the cell grows. Different initial conditions such as constant concentration can of course be tested with equal ease by making the appropriate changes to the model file.

In this example, URDME is invoked in background mode allowing for several parameter cases to be run in parallel on a multicore workstation. Instead of returning the results directly in the workspace, we direct URDME to store the result files and the input files on disk for later post-processing.

### Developing and benchmarking a new algorithm for spatial stochastic simulation

Generally, a large fraction of the effort in developing simulation tools goes into software infrastructure as opposed to code pertaining to the underlying solver algorithms. URDME is designed to provide that infrastructure. The first two layers of the framework provides handling of geometry and meshing, assembly of diffusion jump-rate constants, model integration, pre- and post-processing and data visualization. In this section we will illustrate how to use URDME’s infrastructure to enhance the development and benchmarking of a new stochastic simulation algorithm, DFSP [32]. We will describe the components of this solver and how they are integrated with URDME. This example may therefore serve as a design pattern for algorithm integration into the URDME framework.

Since the diffusion intensity scales differently than the reaction propensities with increasing mesh resolution, diffusion events often occur on a faster time scale than the reactions in the system. Effectively, as the mesh becomes finer a larger and larger percentage of the simulation events will be diffusion jumps. A similar phenomenon, stochastic stiffness, often occurs in simulations of well-stirred models and has led to extensive methods development [37-40]. The DFSP algorithm is an approximate spatial stochastic simulation algorithm which aggregates a large number of diffusive transfers over a time-step. It does this by calculating the probability distribution of a molecule starting in a given voxel after some fixed time-step *τ*_*D*_, and then samples from this distribution to redistribute the molecules. DFSP can in this way give great enhancements in simulation speed at the cost of approximation errors which can be controlled (see [32] for a more extensive analysis).

Integration of a new solver into the URDME framework is designed to be a simple process, with the largest fraction of the required new code being specific to the underlying solver algorithm. URDME solvers have three main components: the solver source code, a Makefile, and an optional pre-execution script. The Makefile creates a standalone Unix executable from the source code. The DFSP solver uses a pre-execution script in Matlab to calculate data specific to the algorithm. This data is then added to the input file that URDME creates upon execution of the solver. Table 1 describes the files that are part of the DFSP solver.

**Table 1:** Overview of the files that make up the DFSP plugin solver

		
**Directory**	**File**	**Description**
urdme/build	Makefile.dfsp	Solver Makefile.
urdme/src/dfsp	dfsp.c	Solver entry point and data initialization.
	dfsp.h	DFSP header file.
	dfspcore.c	Main entry point for the solver.
	dfsp_reactions.c	Simulates reaction events.
	dfsp_diffusion.c	Simulates diffusion events.
urdme/msrc	urdme_init_dfsp.m	Matlab pre-execution script.

This structure shows the design pattern for solver integration into the URDME framework (see [30]).

% DFSP Performance and Error benchmark

% code

tic;

solution = urdme(fem,@fange,{’Solver’, ’nsm’,’Propensities’,’fange’}); nsm_simulation_time = toc

nsm_period= find_mincde_period(solution) for tau_D = [ 0.001, 0.005, 0.01, 0.05, 0.1, 0.5 ]

       tic;

       solution = urdme(fem,@fange,

       {’Propensities’,’fange’,’Solver’,

       ’dfsp’,...

            ’tau’,tau_D,’max_jump’,10,

            ’DFSP_cache’,

             dfsp_cache_filename});

       dfsp_simulation_time = toc

       dfsp_period = find_mincde_period

       (solution)

       error = abs(dfsp_period-nsm_period)/

       nsm_period

end

**Figure 4:**
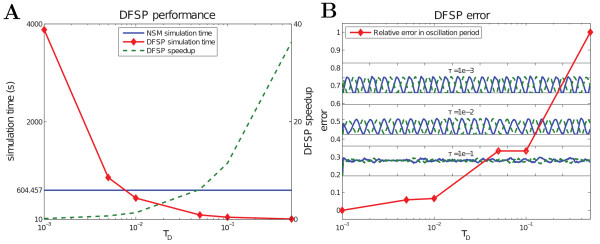
**DFSP benchmark results.** (**A**) Performance of DFSP shows a comparison of simulation times for DFSP at varying *τ*_*D*_values (red) and NSM (blue), and the DFSP speedup factor (green). For this model, DFSP outperforms NSM for *τ*_*D*_>0.01. (**B**) Error in DFSP shows the relative error in MinCDE oscillation period (red) and the oscillation patterns for three simulations. Simulations with *τ*_*D*_<0.1 produces coherent oscillation patterns and result in a negligible error. The system was simulated to a final time 900s. Simulations were performed on a 1.8 Ghz Intel Core i7 processor.

In addition to the lower integration overhead of implementing a new algorithm in the URDME framework, URDME allows developers to easily benchmark their solvers. The code block above shows a Matlab script that sets up a benchmarking experiment to assess the performance and error of the DFSP solver when simulating the model for Min-oscillations described in the first example in this paper. This code also illustrates the calling signature for the function when used with the NSM and DFSP solvers. The DFSP solver takes the additional arguments ‘tau’ as the time-step, ‘max_jump’ as the maximum spatial jump distance, and ‘DFSP_cache’ as the cache file used to store the data specific to the DFSP algorithm. The utility function finds the peak period of the oscillations through straightforward spectral analysis using built-in routines in the Matlab scripting environment, again illustrating the advantage of using the scripting layer’s post-processing capabilities. Figure 4 shows the results of the benchmarking experiment. We find that the DFSP method with 0.01<*τ*_*D*_<0.1 produces simulation results faster than NSM and with good accuracy in the oscillation period.

### Active transport in a neuron

Diffusion is the dominating mechanism of molecular transport in prokaryotes such as *E. Coli*, and it was in that context the NSM was first applied [8,15]. However, diffusion is not the only mechanism for molecular transport in eukaryotic cells. Intra-cellular cargo can be transported by motor proteins along cytoskeletal structures made up of microtubule and actin polymers [41-43]. Molecular motor proteins bind to the cargo and to the filaments and move the cargo along the fiber, always in a specific direction depending on the type of motor and fiber. This transport is usually much faster than diffusion but requires an energy input. Vesicles, organelles, mRNA and proteins involved in signaling are examples of cargo that are transported in this way inside living cells.

Due to the ubiquity of active transport in biological systems, it is important that simulation software have the capability to handle mesoscopic models with general transport mechanisms. In [35], the RDME was extended to include an advection term that models cargo transport on the microtubule network. A simple model of signaling in a yeast cell was considered and URDME was used for model development and simulation. To illustrate both the geometrical flexibility of URDME as well as its capability to model more general transport mechanisms, we show here how to simulate active transport in a model of a neuron with a detailed geometry.

Active transport of cellular cargo is of fundamental importance to maintain the highly polarized state of a healthy neuron. In the axon, microtubules are uniformly oriented with plus-end towards the soma and minus-end towards the synapse. Kinesin transports cargo in the anterograde direction, from the cell body to the synapse. For example, kinesin drive the transport of synaptic vesicles from the cell body through the axon where they are subsequently docked to the plasma membrane in the presynaptic terminus. Dynein drives transport in the opposite direction (retrograde transport) in the axon, and may aid in transporting for example RNA from the cell body to the dendrites [44]. In the dendrites, the situation is more complex than in the axon, since the microtubules form an array of mixed orientation. While the particular motor protein transports cargo in a specific direction on the fibers, a single cargo such as a vesicle can have many different motors bound to it simultaneously and therefore may move in a bidirectional manner [45-47]. The details of how kinesin and dynein-driven transport is coordinated and regulated to achieve differential targeting and localization of cargo is still a largely unresolved issue [48,49]. As an example of a possible mechanism of regulation, the microtubule binding protein Tau effects the binding affinity of kinesin to the microtubule, while dynein is less sensitive to elevated Tau concentrations [50].

**Figure 5:**
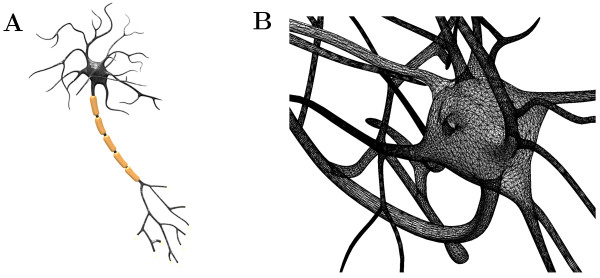
**Geometry and mesh for a model of a neuron.** The neuron geometry (**A**) is based on a artistic CAD rendering generated with the public domain version of the software Blender (http://www.blender.org). In order to conduct simulations in this geometry, the model was exported in the STL surface mesh format, imported into the open-source meshing package Gmsh [31], where the boundary was re-parametrized and the domain subsequently meshed with a volume mesh in 3D. The resulting mesh was then converted into a Comsol Multiphysics 3.5a model to serve as a geometry description for the URDME model. Assembly of active transport jump rate constants are conducted by URDME on the unstructured mesh shown in (**B**). For a mathematical background on how to obtain these constants on the unstructured mesh, see [35]. URDME’s capability to use an unstructured mesh made up of tetrahedral and triangular elements is of vital importance in order to be able to resolve the complex geometry of the neuron.

To illustrate how diffusion and active transport can simultaneously be modeled with URDME in the neuron geometry shown in Figure 5 we consider a straightforward model where a cargo species is transported to different regions of the neuron. The motor proteins are modeled *implicitly*, that is, we assume that a population of motor proteins is associated to the cargo species at all times. Although an approximation, there are recent experimental evidence that the distributions of motors on vesicles are relatively stable [47]. Table 2 summarizes the model. Here, the cargo species *V * is created uniformly in the cell body (R1). *V * can diffuse and bind reversibly to microtubule filaments, either with a kinesin motor as ^*Vk*^ or with a dynein motor as ^*Vd*^(R2–R5). When bound to a filament, *V * is actively transported in a direction dictated by the kind of motor that is currently active. The cargo can reverse its direction on the fiber in bidirectional transport by letting the currently active motor protein change with some probability (R6,R7). The quotient *σ*_*kd*_/*σ*_*dk*_ then dictates the direction of net transport. Finally, *V * is uniformly degraded (R8) in the whole neuron so that the total number of cargo *V * reaches a steady-state level.

**Table 2:** Reactions of the transport model

			
	**Reaction**	**Description**	**Cellular location**
(R1)	$\emptyset \overset{\mu_{1}}{\underset{}{\to }}V$	Creation of cargo	Cell body
(R2–R5)	$V\overset{\sigma_{b}}{\underset{\sigma_{d}}{⇌}}V^{k,d}$	Binding of *V* to microtubule	All domains
(R6,R7)	$V^{k}\overset{\sigma_{\mathrm{kd}}}{\underset{\sigma_{\mathrm{dk}}}{⇌}}V^{d}$	Reversal of direction	Microtubule
(R8)	$V\overset{\mu_{2}}{\underset{}{\to }}\emptyset$	Degradation of *V*	All domains
			

Model of active transport of a cargo species *V* that is transported on microtubule filaments in a direction determined by the orientation of the fibers (as modeled by a velocity field) and the current motor protein bound to the fiber (kinesin or dynein).

To illustrate the ability of cargo to localize to different compartments of the cell depending on the dominating motor protein we consider the following scenario. First, we let *σ*_*dk*_=10*σ*_*kd*_, so that on the average, kinesin will spend more time bound to the microtubule than dynein will do. In this case, the cargo will travel through the axon and eventually localize to the axon terminus. After half of the total simulation time has elapsed, the situation is reversed and *σ*_*kd*_=10*σ*_*dk*_such that the cargo will localize to the dendrites.

**Figure 6:**
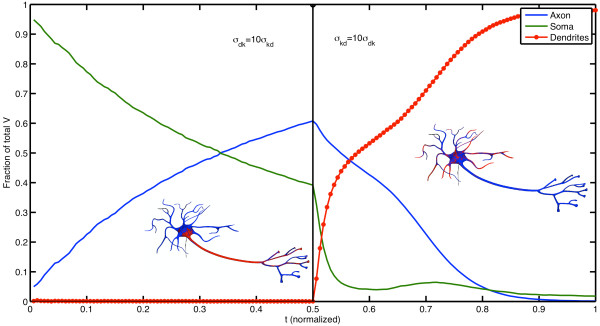
**Localization as a function of the binding rates to microtubules for the different motor proteins.** Normalized concentration of *V * in the soma (green), axon (blue) and in the dendrites (red) as a function of time. Initially, the parameters satisfy *σ*_*kd*_=10*σ*_*dk*_and cargo localizes to the axon due to the larger fraction of time spent in the kinesin binding state. At time *t*=0.5 the situation is reversed, and the localization of *V * shifts from axon to dendrites. The red regions in the inlays depicting the neuron shows the areas where *V * is present.

Figure 6 shows a typical output of a simulation with URDME. The fraction of the total number of cargo *V * is plotted in the different regions of the neuron geometry (axon, soma, and dendrites). Since the purpose of this example is to illustrate the capability of URDME to model both diffusion and active transport in a complex geometry, the values of the various parameters have not been chosen to fit any particular neuron geometry. Hence the velocity of dynein is conveniently set to be half of that of kinesin in the axon. Also, the net rate of transport in the dynein is set to be one hundredth of the rate of kinesin in the axon to reflect the effects of mixed polarity of fibers [51].

In order to setup this simulation in URDME, a Matlab function for the velocity field modeling the average orientation of the fibers at any point in the domain needs to be provided. Obviously, specification of this velocity field requires biological knowledge. The ability to work in the Matlab environment greatly simplifies parametrization of the velocity field. Since this geometry was given as a surface mesh, which is also often the case when the domain is obtained from cell imaging, we have no analytical expression for the parametrization of the geometry to rely on. In this example we want the velocity field to trace the axon and dendrite structures. To achieve this, we first compute surface normals to all triangles on the surface of the neuron. An interpolation table containing vectors with base in the centroids in the triangles of the surface mesh and pointing in the direction of suitably chosen reference points was thus constructed. For simplicity, we only used two different reference points, one near the center of the cell body and the other beyond the axon terminus along the long axis of the axon. The smoothness of the velocity field can easily be improved by adding more reference points. For any point inside the domain, we evaluate the velocity by nearest neighbor interpolation using the interpolation table. From this description of the microtubule network and the information about the mesh, utility routines available as add-ons to the basic URDME package can be used to assemble jump rate constants to be used in the definition of the stochastic transport process in much the same way as for diffusion [35]. This procedure may seem complicated at a first glance, but can be performed quite easily in Matlab using built-in utility routines. The model files required to run this example can be found in Additional file 4.

## Discussion

The design of URDME is motivated by both modeling and algorithm development. Systems biology investigations are typically computational intensive, and often require large ensembles of trajectories spanning parameter space to match data, or to conduct a sensitivity and robustness analysis.

Development of more efficient simulation methods is needed to make such large scale investigations feasible. However, due to the overhead of handling complex geometries, mesh generation and visualization of results, algorithm developers often tend to consider only simple test models in simple geometries, often restricted to one or two spatial dimensions. While this can be enough to illustrate the potential benefits of a new method, the resulting software is often not general enough for use on complex biological models. URDME aims to bridge this gap by facilitating for method developers by providing a large part of the infrastructure needed for simulation of realistic models. We exemplified this in the paper by the extension of the approximate algorithm DFSP to a full 3D simulation.

The theory and methodology for spatial stochastic simulation is still undergoing extensive development, and no single mathematical modeling framework or method has emerged as a *de facto* standard. The utility of the URDME framework is not restricted to mesoscopic RDME simulations; we have used URDME to develop solvers based on the Smoluchowski model and a microscopic–mesoscopic hybrid methods [34].

Another benefit of the modular architecture is that it simplifies the use of different execution models for the simulations. As part of work on methods for enactment of computation in grid environments, we are developing a URDME server module that enables remote execution in distributed computing environments [52]. This enables highly task-parallel investigations to utilize distributed computational resources such as clusters, grids, and clouds to greatly increase productivity for the end-user.

### Comparison of spatial stochastic software packages

To further illustrate the design of our software, we have compared its features to two other publicly available packages for mesoscopic spatial stochastic simulation. Table 3 shows a comparison between URDME 1.1, MesoRD 1.0, and STEPS 1.3. MesoRD was one of the first software projects aimed at simulation of the RDME. STEPS was developed for simulation of detailed models of dendrites and synapses, but is generally applicable to a lager set of reaction-diffusion models.

**Table 3:** A comparison of features of RDME simulation software

			
	**URDME 1.1**	**MesoRD 1.0**	**STEPS 1.3**
Interface	Matlab & Comsol	Command line	Python
		Simple GUI (Windows)	
Visualization	Matlab & Comsol	OpenGL tool	PyOpenGL tool
		Matlab toolbox	
Post-processing	Matlab	3rd party	Python
SBML support	Conversion tool	SBML L2v4	Import module
	(no geometry)	+ CSG geometry	(no geometry)
Edit Geometry	Comsol	SBML	3rd party
Mesh Type	Vertex centered	Uniform Cartesian	Body centered
	Tetrahedrons		Tetrahedrons
Algorithms	NSM, DFSP	NSM	Spatial-SSA
	+ extendable	+non-local extension	
Propensity types	All	SBML (MathML)	Mass-action
Model Features	compartments	compartments	compartments
	surfaces		surfaces
	volume diffusion	volume diffusion	volume diffusion
	surface diffusion		
	directed transport		

This table summarizes the main differences and similarities between the software packages URDME, MesoRD and STEPS.

There are three significant ways a user interacts with a spatial stochastic software package: the environment for model development, execution of a simulation, and post-processing and analysis of the data generated by the simulation.

The interface and model development environment used by URDME and STEPS are similar in that both are closely tied to a programming language environment: Matlab in the case of URDME and Python for STEPS. URDME provides a single function entry point, and models are developed in external programming files. This design pattern follows that of the Matlab ODE suite. STEPS provides an object oriented Python interface for creation, simulation and post-processing of models. STEPS claims that a programmatic interface offers significant advantages over non-interactive software interface [22] (in contrast to the command line and input file interface), and we share this opinion.

The major differences between URDME and STEPS are the feature set and the performance. The execution platform of URDME is the Matlab-Comsol environment, thus URDME has full access to the scientific libraries of Matlab as well as the advanced geometry and mesh handling interface of Comsol. Another major difference is one of aim. URDME is developed by a team of biological model developers as well as of algorithm developers, and it aims itself at both communities. This is reflected in its expandable solver interface and performance centric design.

In contrast to the design pattern used in URDME and STEPS, MesoRD functions as a command line program that uses an input file in the Systems Biology Markup Language (SBML) [53] format to describe the model. SBML is a community effort with the aim to standardize descriptions of biochemical reaction network models. MesoRD extends the format with a custom Consecutive Solid Geometry (CSG) description of the domain geometry of the model. SBML has been widely adopted as a standard to exchange non-spatial models, but the limitations in its capability to describe spatial models has restricted its adoption for RDME simulations.

The post-processing environment of URDME is closely integrated into Matlab. MesoRD provides a Matlab toolbox for analyzing the simulation data files. STEPS utilizes the Python programming environment and packages such as NumPy, SciPy, and Matplotlib for post-processing and analysis.

Compared to static XML input files, the programmatic paradigm used by URDME and STEPS provides a more powerful but also more complex modeling environment. Constructing model files using a complete programming language reduces the restrictions imposed on the software by the model format. For example, the model of the neuron presented in the Results section could not have been described by an SBML document, nor the extended SBML format used by MesoRD. Since propensities in URDME are defined in a program file, any type of functional propensity can be used in URDME models, including Michaelis-Menten and Hill term style propensities, and even arbitrary logical expressions can be employed.

This offers great flexibility in terms of the models that can be simulated, but also places more responsibility on the end-user. MesoRD uses MathML as part of the SBML definition, which allows the use of any mathematical expression in the propensities and facilitates handling of units and error checking. This is a powerful and robust, but also a computationally very expensive strategy. The STEPS reaction object only supports mass action kinetics, which results in an efficient but less flexible strategy.

In addition to having the most efficient and expandable design of the model propensity, URDME also provides the largest set of geometry and mesh model features of the three software packages. URDME supports volume compartments with internal and external 2D surfaces embedded in the 3D geometry, as well as diffusion and reactions on surfaces and in the 3D volume. URDME also supports directed transport (convection) in 3D through an add-on module. STEPS 1.3 supports 3D compartments and volume diffusion. It is capable of localizing species to a curved surface embedded in 3D, but does not support surface diffusion. MesoRD 1.0 supports 3D compartments and volume diffusion only. To represent cellular membranes, their models typically use a small 3D volume on the exterior of the domain.

In summary, as a consequence of the design of the model environment, MesoRD is simpler to learn and use than both URDME and STEPS and also offers a better support for e.g. handling units, but the latter two offer a much more flexible and efficient modeling and simulation environment. In addition to the programmatic environment, both URDME and STEPS provide limited support for SBML. URDME has an experimental conversion utility that will create templates for the model and propensity file from an SBML description of the chemical reactions, see Additional file 5. This utility will be fully included in the next version of URDME. STEPS provides a function to convert an SBML file into Python model objects. In addition to the SBML document defining the biochemical reaction network, both URDME and STEPS require a mesh describing the model domain geometry be provided.

### Simulation performance

**Figure 7:**
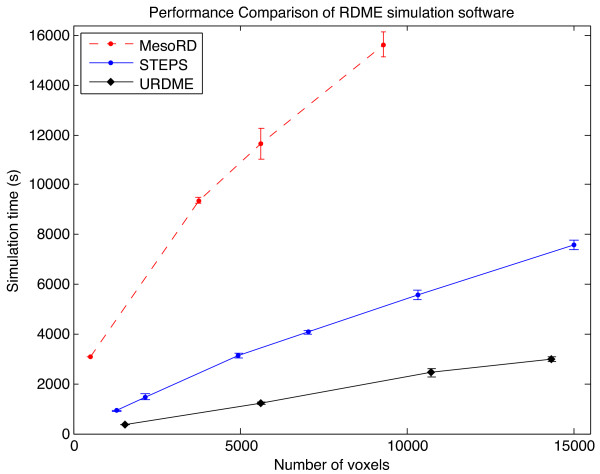
**Performance comparison of the three software packages for an increasing number of voxels.** Each point shows the mean and the error bars show the standard deviation of a ensemble of *N*=5 runs. For URDME the number of voxels represents the number of mesh vertices, for MesoRD it represents the number of cubic subvolumes, and for STEPS it represents the number of tetrahedrons. All simulations were performed on a 1.8 GHz Intel core i7 processor with 4GB of memory.

To compare the performance of the software packages, we implemented the model of Min oscillations in *E. Coli* as described in [8] in each of the three software environments. Figure 7 shows simulation time as a function of the number of voxels in the mesh. The simulation was run for 900 seconds (simulation time), with the system state recorded every second. A detailed description of the model setup in the different packages can be found in Additional file 6 and the scripts used for producing these benchmarks are provided in Additional file 7. The URDME framework has a strong emphasis on efficient simulation algorithms which is also visible in the figure. URDME clearly outperforms the other packages and we believe that this is in large parts due to URDME’s modular design and the fact that the solver source files and the propensity functions file are *compiled* into a dedicated executable for each separate model (see the Implementation section for details).

### The numerical treatment of mesoscopic diffusion

URDME emphasizes the use of unstructured tetrahedral and triangular meshes to discretize the geometry. Unstructured meshes offer distinct advantages over Cartesian meshes for resolving complex geometries with non-trivial boundaries and they are more flexible than cut-cell approaches when it comes to describing processes occurring on a curved boundary embedded in 3D space, such as the cell membrane of a spherical cell or the nuclear membrane [54]. The first version of URDME was developed as a product of theoretical work on how to obtain mesoscopic diffusion jump constants on triangular and tetrahedral meshes [33]. In short, the methodology used by URDME is based on the fact that a numerical discretization scheme for the standard diffusion equation will give jump coefficients that result in mesoscopic simulations that are consistent with both the behavior of mean values of a large ensemble of particles and the probability density function for a single particle diffusing according to Brownian motion. The latter is true since the Fokker-Planck equation for the one-particle probability density function is mathematically equivalent to the macroscopic diffusion equation. URDME currently uses a discretization with the Finite Element method to obtain the diffusion jump coefficients.

The quality of the tetrahedral mesh is an important aspect of a numerical discretization. An in-depth discussion of the requirements on the mesh for use in the mesoscopic model is given in [33]. Tetrahedra should not be too irregular, and between regions in the domain with much different resolution, the size of the elements should not grow too fast. This is also true for the solution of PDEs, and mesh generation software is aware of these issues and attempts to optimize the meshes accordingly. Surface meshes in 2D from state-of-the art mesh generation software such as Comsol tend to be of very high quality. In 3D, many meshes will violate the assumptions in [33] to some degree. Generation of high quality unstructured meshes is an active area of research due to their importance in industrial applications. The modular design of URDME ensures that we can accommodate new results in this area without major restructuring of the code.

The influence of mesh quality on RDME simulations on unstructured meshes in 3D was studied for several different discretization schemes in [55] using particularly revealing and highly sensitive model problems. They show that unless the meshes are of high quality, discretization errors may lead to small but persisting errors for both the Finite Element and the Finite Volume methods, i.e. the convergence properties of the schemes are affected negatively. In some of these cases, simulations using a structured Cartesian mesh will have better numerical properties if the geometry permits resolution of the domain with a feasible number of subvolumes. On the other hand, it is not difficult to think of cases for which this is very difficult and for which sensitive processes occur on the parts of the domain which are hard to resolve.

Using MesoRD, surfaces in a 3D model are modeled as volume geometry objects by ensuring that the thickness of the membrane is small compared to its size, approaching a true 2D model as the thickness of the membrane becomes small. Unless one desires to resolve some dynamics on such high level of detail as to consider vertical movement of molecules in the membrane, this will be unnecessarily expensive since the mesh elements has to be sufficiently small to resolve the narrow 3D volume. The mesh generation in MesoRD needs several grid points in the extent of a membrane to give a fully connected diffusion volume [56](Figure eleven). With a uniform grid, this will lead to expensive simulations since the size of the voxels necessary to accurately resolve the membrane must be used everywhere in the domain. In order to demonstrate this, we conducted a simple diffusion-only numerical experiment, described in detail in Additional file 6. We let molecules diffuse freely on the surface of a unit sphere, and be absorbed by a small circular patch at one of the poles. Simulations using URDME are in excellent agreement with the exact solution even for fairly coarse meshes (Additional file 6: Figure S1). For example, using an ensemble size of 1^05^ molecules to compute the mean absorption time, the error was ≈0.2*%*for a mesh with 4343 voxels. The computing time to generate the solution was 21 seconds. By contrast, for a membrane thickness of 100 *nm* and a voxel size of 20 *nm*, MesoRD 1.0 produces a solution with about 14% error using 157128 voxels and a simulation time of 1 hour and 50 minutes on the same 2.66 Ghz Intel Core i7 with 8GB of RAM.

For complex models with both volume diffusion, surface diffusion, and reactions, it is difficult to predict what impact different sources of error in the diffusion will have on the output metric of interest. For example, for the Min system used to benchmark the different software packages in Figure 7, URDME, STEPS, and MesoRD give quite similar period times of oscillations (Additional file 6).

In addition to errors caused by the discretization, errors intrinsic to the RDME mathematical model arise for highly diffusion limited reactions when the voxels become very small [12]. To some extent, this can be alleviated using modified, mesh dependent bimolecular reaction rates [13,57], but there is a critical size of the voxels under which no correction to the traditional RDME can make it consistent with more fine scaled particle based methods [58]. Since unstructured meshes can more accurately resolve complex geometries, their spatial accuracy is often higher for equivalently sized voxels when compared to Cartesian meshes. This can help in avoiding geometrical features of the model to force us to approach the critical regime for the voxel sizes. The combined effects of diffusion discretization error and error caused by small subvolumes were investigated for several additional models in [55]. For the examples studied there, it was concluded that the error introduced by small subvolumes in 3D could be a bigger source of error than any numerical discretization errors of the diffusion operator.

## Conclusions

As demonstrated by the examples in this paper, the URDME infrastructure offers great flexibility at the stage of model construction and execution. Using a simple script in Matlab, URDME was used to set up and conduct a series of experiments in which the geometry of an *E. Coli* bacterium was automatically varied. In another example, the basic reaction-diffusion modeling framework was extended to include active transport in a highly complex geometry obtained from external CAD and meshing software.

The URDME software framework offers unique features for both model and methods developers in computational systems biology. The support of unstructured meshes provides the capability to create models with a complex geometry that closely match the physical descriptions of the systems under study. URDME integrates easily with widely used scientific computing software to provide a versatile platform for mathematical and computational modeling, allowing for the implementation of complex and customized models and pre- and post-processing routines. The modular design ensures extensibility and interchangeability of the third-party tools used for model specification and mesh generation, as well as of the core simulation algorithms.

## Availability and requirements

· Project name: URDME.

· Project home page: http://www.urdme.org.

· Operating systems: Linux, MacOS X.

· Programming language: C, Matlab, Bash shell script.

· Other requirements: GNU GCC version ≥ 4.2, Matlab, Comsol Multiphysics 3.5a.

· License: GNU General Public License, version 3.

· Any restrictions to use by non-academics: none.

## Competing interests

The authors declare that they have no competing interests.

## Authors’ contributions

BD, SE and AH contributed equally to the design and implementation of the software and to the preparation of this paper. All authors read and approved the final manuscript.

## Funding

The Swedish Graduate School in Mathematics and Computing (AH, SE), the Swedish Research Council and the Linnaeus centre of excellence UPMARC (SE), the Royal Swedish Academy of Sciences FOA08H-109, FOA09H-63, FOA09H-64 (AH), SSF grant A3 02:124 (AH), U.S. NSF grant DMS-1001012, U.S DOE award DE-FG02-04ER25621, U.S. NSF IGERT DGE-02-21715, Institute for Collaborative Biotechnologies grant W911NF-09-0001 from the U.S. Army Research Office (BD) and the National Institute of Biomedial Imaging And Bioengineering of the National Institute of Health under Award Number R01 EB014877 (BD,AH). The content is solely the responsibility of the authors and does not necessarily represent the official views of the National Institute of Health.

## Supplementary Material

Additional file 1 **urdme.tar.gz.** The current release of URDME. Also available for download via http://www.urdme.org. Click here for file

Additional file 2 **minsweep.tar.gz.** Model files required to run the first example in the main paper. Click here for file

Additional file 3 **benchmark.tar.gz.** Model files required to run the second example in the main paper. Click here for file

Additional file 4 **neuron.tar.gz.** Model files required to run the third example in the main paper. Click here for file

Additional file 5 **urdme_sbml_converter.tar.gz.** SBML conversion tool to create URDME model files from a SBML model file describing chemical reactions. Click here for file

Additional file 6 **validation.pdf.** Simulation results for a simple diffusion problem on the surface of a sphere and for the Min system [8,34,55,56,59,60]. Click here for file

Additional file 7 **urdme_software_comparision.tar.gz.** Model files and scripts used to conduct the performance benchmark in the discussion section and the simulations described in Additional file 6. Click here for file

## References

[B1] Thattai M van Oudenaarden A Intrinsic noise in gene regulatory networks Proc Natl Acad Sci USA 2001 98 8614 8619 10.1073/pnas.151588598 11438714 PMC37484

[B2] Paulsson J Berg OG Ehrenberg M Stochastic focusing: Fluctuation-enhanced sensitivity of intracellular regulation Proc Natl Acad Sci USA 2000 97 13 7148 7153 [http://www.pnas.org/cgi/content/abstract/97/13/7148]. 10.1073/pnas.110057697 10852944 PMC16514

[B3] Barkai N Leibler S Circadian clocks limited by noise Nature 2000 403 267 268 10659837 10.1038/35002258

[B4] Swain PS Elowitz MB Siggia ED Intrinsic and extrinsic contributions to stochasticity in gene expression Proc Natl Acad Sci USA 2002 99 20 12795 12800 10.1073/pnas.162041399 12237400 PMC130539

[B5] Elowitz MB Levine AJ Siggia ED Swain PS Stochastic gene expression in a single cell Science 2002 297 5584 1183 1186 [http://www.sciencemag.org/cgi/content/abstract/297/5584/1183]. 10.1126/science.1070919 12183631

[B6] Raser JM O’Shea EK Noise in gene expression: Origins, consequences and control Science 2005 309 2010 2013 10.1126/science.1105891 16179466 PMC1360161

[B7] Gillespie DT A general method for numerically simulating the stochastic time evolution of coupled chemical reacting systems J Comput Phys 1976 22 403 434 10.1016/0021-9991(76)90041-3

[B8] Fange D Elf J Noise induced Min phenotypes in E. coli. PLoS Comput Biol 2006 2 6 e80 10.1371/journal.pcbi.0020080 PMC1484588 16846247

[B9] Takahashi K Tănase-Nicola S ten Wolde PR Spatio-temporal correlations can drastically change the response of a MAPK pathway Proc Natl Acad Sci USA 2010 107 6 2473 2478 10.1073/pnas.0906885107 20133748 PMC2811204

[B10] van Kampen NG Stochastic Processes in Physics and Chemistry. 3rd edition 2007 Amsterdam: Elsevier

[B11] Gardiner CW Handbook of Stochastic Methods for Physics, Chemistry and the Natural Sciences 3rd edition 2004 Berlin: Springer-Verlag

[B12] Isaacson SA The reaction-diffusion master equation as an asymptotic approximation of diffusion to a small target SIAM J Appl Math 2009 70 77 111 10.1137/070705039

[B13] Erban R Chapman J Stochastic modelling of reaction-diffusion processes: Algorithms for bimolecular reactions Phys Biol 2009 6 046001 10.1088/1478-3975/6/4/046001 19700812

[B14] Bernstein D Simulating mesoscopic reaction-diffusion systems using the Gillespie algorithm Phys Rev E 2005 71 041103 10.1103/PhysRevE.71.041103 15903653

[B15] Elf J Ehrenberg M Spontaneous Separation of bi-stable biochemical systems into spatial domains of opposite phases Syst Biol 2004 1 2 230 236 10.1049/sb:20045021 17051695

[B16] Stundzia AB Lumsden CL Stochastic simulation of coupled reaction-diffusion processes J Comput Phys 1996 127 196 207 10.1006/jcph.1996.0168

[B17] Raj A van den Bogaard Rifkin SA van Oudenaarden A Imaging individual mRNA molecules using multiple singly labeled probes Nat Meth 2008 5 10 877 879 10.1038/nmeth.1253 PMC3126653 18806792

[B18] Elf J Li GW Xie XS Probing Transcription factor dynamics at the single-molecule level in a living cell Science 2007 316 5828 1191 1194 10.1126/science.1141967 17525339 PMC2853898

[B19] van Zon JS ten Wolde RP Simulating biochemical networks at the particle level and in time and space: green’s function reaction dynamics Phys Rev Lett 2005 94 12 128103 15903966 10.1103/PhysRevLett.94.128103

[B20] Andrews SS Addy NJ Brent R Arkin AP Detailed simulations of cell biology with Smoldyn 2.1 PLoS Comput Biol 2010 6 3 e1000705 10.1371/journal.pcbi.1000705 20300644 PMC2837389

[B21] Kerr RA Bartol TM Kaminsky B Dittrich M Chang JCJ Baden SB Sejnowski TJ Stiles JR Fast Monte Carlo simulation methods for biological reaction-diffusion systems in solution and on surfaces SIAM J Sci Comput 2008 30 6 3126 3149 10.1137/070692017 20151023 PMC2819163

[B22] Hepburn I Chen W Wils S Schutter ED STEPS: efficient simulation of stochastic reaction-diffusion models in realistic morphologies BMC Syst Biol 2012 6 35 [http://www.biomedcentral.com/1752-0509/6/36/abstract]. 10.1186/1752-0509-6-36 PMC3472240 22574658

[B23] Lampoudi S Gillespie DT Petzold LR The multinomial simulation algorithm for discrete stochastic simulation of reaction-diffusion systems J Chem Phys 2009 130 9 094104 [http://link.aip.org/link/?JCP/130/094104/1]. 10.1063/1.3074302 19275393 PMC2671688

[B24] Rossinelli D Bayati B Koumoutsakos P Accelerated stochastic and hybrid method for spatial simulations of reaction-diffusion systems Chem Phys Lett 2008 451 136 140 10.1016/j.cplett.2007.11.055

[B25] Marquez-Lago TT Burrage K Binomial tau-leap spatial stochastic simulation algorithm for applications in chemical kinetics J Chem Phys 2007 127 10 104101 [http://link.aip.org/link/?JCP/127/104101/1]. 10.1063/1.2771548 17867731

[B26] Bayati B Chatelin P Koumoutsakos P Adaptive mesh refinement for stochastic reaction-diffusion processes J Comput Phys 2011 230 13 26 10.1016/j.jcp.2010.08.035

[B27] Ferm L Hellander A Lötstedt P An adaptive algorithm for simulation of stochastic reaction-diffusion processes J Comput Phys 2010 229 2 343 360 10.1016/j.jcp.2009.09.030

[B28] Hattne J Fange D Elf J Stochastic reaction–diffusion simulation with MesoRD Bioinformatics 2005 21 12 2923 2924 10.1093/bioinformatics/bti431 15817692

[B29] Ander M Beltrao P Ventura BD Ferkinghoff-Borg J Foglierini M Lemerle C Tomas-Oliveira I Serrano L SmartCell, a framework to simulate cellular processes that combines stochastic approximation with diffusion and localisation: analysis of simple networks Syst Biol 2004 1 129 138 10.1049/sb:20045017 17052123

[B30] Drawert B Engblom S Hellander A URDME 1.1: User’s manual Tech. Rep. 2011-003, Department of Information Technology 2011 Division of Scientific Computing

[B31] Geuzaine C Remacle JF Gmsh: a three-dimensional finite element mesh generator with built-in pre- and post-processing facilities [http://www.geuz.org/gmsh/].

[B32] Drawert B Lawson MJ Petzold L Khammash M The diffusive finite state projection algorithm for efficient simulation of the stochastic reaction-diffusion master equation J Chem Phys 2010 132 7 074101 10.1063/1.3310809 20170209 PMC2905448

[B33] Engblom S Ferm L Hellander A Lötstedt P Simulation of stochastic reaction–diffusion processes on unstructured meshes SIAM J Sci Comput 2009 31 3 1774 1797 10.1137/080721388

[B34] Hellander A Hellander S Lötstedt P Coupled mesoscopic and microscopic simulation of reaction-diffusion processes in mixed dimensions Multiscale Model Simul 2012 10 2 585 611 10.1137/110832148

[B35] Hellander A Lötstedt P Incorporating active transport in mesoscopic reaction-transport models inside living cells Multiscale Model Simul 2010 8 5 1691 1714 10.1137/100784709

[B36] Huang KC Meir Y Wingreen NS Dynamic structures in Escherichia coli: Spontaneous formation of MinE and MinD polar zones Proc Natl Acad Sci USA 2003 100 22 12724 12728 10.1073/pnas.2135445100 14569005 PMC240685

[B37] Rathinam M Petzold L Cao Y Gillespie D Stiffness in stochastic chemically reacting systems: The implicit tau-leaping method J Chem Phys 2003 119 24 12784 12794 http://link.aip.org/link/?JCP/119/12784/1]. 10.1063/1.1627296

[B38] Cao Y Gillespie D Petzold L The slow-scale stochastic simulation algorithm J Chem Phys 2005 122 014116 http://link.aip.org/link/?JCP/122/014116/1]. 10.1063/1.1824902 15638651

[B39] Cao Y Petzold L Slow scale Tau-Leaping method Comput Meth Appl Mech Eng 2008 197 3472 3479 [PCMID:PMC2753989] 10.1016/j.cma.2008.02.024 PMC2753989 19794828

[B40] Wu S Fu J Cao Y Petzold L Michaelis–Menten speeds up tau-leaping under a wide range of conditions J Chem Phys 2011 134 13 134112 [http://link.aip.org/link/?JCP/134/134112/1]. 10.1063/1.3576123 21476748 PMC3087420

[B41] Howard J The movement of kinesin along microtubules Annu Rev Physiol 1996 58 703 729 10.1146/annurev.ph.58.030196.003415 8815816

[B42] Mallik R Gross SP Molecular motors: strategies to get along Curr Biol 2004 14 971 982 10.1016/j.cub.2004.10.046 15556858

[B43] Vale RD The molecular motor toolbox for intracellular transport Cell 2003 112 467 480 10.1016/S0092-8674(03)00111-9 12600311

[B44] Martin KC Zukin RS RNA trafficking and local protein synthesis in dendrites: an overview J Neurosci 2006 26 27 7131 7134 10.1523/JNEUROSCI.1801-06.2006 16822966 PMC6673931

[B45] Welte MA Bidirectional transport along microtubules Curr Biol 2004 14 525 537 10.1016/j.cub.2004.06.045 15242636

[B46] Gross SP Vershinin M Shubeita GT Cargo transport: two motors are sometimes better than one Curr Biol 2007 17 R478—R486 17580082 10.1016/j.cub.2007.04.025

[B47] Encalada SE Szpankowski L Xia Ch Goldstein LSB Stable kinesin and dynein assemblies drive the axonal transport of mammalian prion protein vesicles Cell 2011 144 4 551 565 http://linkinghub.elsevier.com/retrieve/pii/S0092867411000602]. 10.1016/j.cell.2011.01.021 21335237 PMC3576050

[B48] Goldstein LSB Yang Z Microtubule-based transport systems in neurons: the roles of kinesins and dyneins Ann Rev Neurosci 2000 23 39 71 [http://www.annualreviews.org/doi/abs/10.1146/annurev.neuro.2 3.1.39]. 10.1146/annurev.neuro.23.1.39 10845058

[B49] Schlager MA Hoogenraad CC Basic mechanisms for recognition and transport of synaptic cargos Mol Brain 2009 2 25 [http://www.molecularbrain.com/content/2/1/25]. 10.1186/1756-6606-2-25 PMC2732917 19653898

[B50] Dixit R Ross JL Goldman YE Holzbaur EF Differential regulation of dynein and kinesin motor proteins by Tau Science 2008 319 5866 1086 1089 10.1126/science.1152993 18202255 PMC2866193

[B51] Hirokawa N Takemura R Molecular motors and mechanisms of directional transport in neurons Nat Rev Neurosci 2005 6 3 201 214 10.1038/nrn1624 15711600

[B52] Östberg PO Hellander A Drawert B Elmroth E Holmgren S Petzold L Reducing complexity in management of scientific computations Proceedings of CCGrid 2012 - The 12th IEEE/ACM International Symposium on Cluster, Cloud and Grid Computing 2012 845 852

[B53] Hucka M Finney A Sauro HM Bolouri H Doyle JC H K the rest of the SBML Forum: Arkin AP Bornstein BJ Bray D Cornish-Bowden A Cuellar AA Dronov S Gilles ED Ginkel M Gor V Goryanin II Hedley WJ Hodgman TC Hofmeyr JH Hunter PJ Juty NS Kasberger JL Kremling A Kummer U Le Novere N Loew LM Lucio D Mendes P Minch E Mjolsness ED Nakayama Y Nelson MR Nielsen PF Sakurada T Schaff JC Shapiro BE Shimizu TS Spence HD Stelling J Takahashi K Tomita M Wagner J Wang J The systems biology markup language (SBML): a medium for representation and exchange of biochemical network models Bioinformatics 2003 19 4 524 531 10.1093/bioinformatics/btg015 12611808

[B54] Isaacson SA Peskin CS Incorporating diffusion in complex geometries into stochastic chemical kinetics simulations SIAM J Sci Comput 2006 28 47 74 10.1137/040605060

[B55] Kieri E Accuracy aspects of the reaction-diffusion master equation on unstructured meshes Master’s thesis 2010 Department of Information Technology, Uppsala University

[B56] Elversson D Kieri E Spatial stochastic simulation of Spatial stochastic simulation of Spatial stochastic simulation of cellular reaction networks: A comparison of discretizations of the Laplace operator for mesoscopic diffusion Master’s thesis 2010 Department of Information Technology, Uppsala University

[B57] Fange D Berg OG Sjöberg P Elf J Stochastic reaction-diffusion kinetics in the microscopic limit Proc Natl Acad Sci USA 2010 107 46 19820 19825 10.1073/pnas.1006565107 21041672 PMC2993376

[B58] Hellander S Hellander A Petzold L On the reaction-diffusion master equation in the microscopic limit Phys Rev E 2012 85 4 042901 10.1103/PhysRevE.85.042901 22680526

[B59] Bloomfield VA Prager S Diffusion-controlled reactions on spherical surfaces Biophys J 1979 27 447 453 10.1016/S0006-3495(79)85228-5 262439 PMC1328599

[B60] Lindermann J Lauffenburger D Analysis of intracellular receptor/ligand sorting-calculation of mean surface and bulk diffusion times within a sphere Biophys J 1986 50 295 305 10.1016/S0006-3495(86)83463-4 3741985 PMC1329746

