# Supplementary material for: URDME: a modular framework for stochastic simulation of reaction-transport processes in complex geometries
Source: BMC Syst Biol. 2012 Jun 22;6:76. doi: 10.1186/1752-0509-6-76 (PMC3439286; doi:10.1186/1752-0509-6-76)
Supplement: Additional file 1 — urdme.tar.gz. The current release of URDME. Also available for download via http://www.urdme.org. [file 1752-0509-6-76-S1.gz › urdme-1.1.2a/urdme/doc/manual.pdf]

URDME *v.* 1.1: User's manual

Josef Cullhed, Brian Drawert[2], Stefan Engblom[1], Andreas Hellander[1,*]

July 26, 2010

[1] *Div of Scientific Computing, Dept of Information Technology*
Uppsala University, P. O. Box 337, SE-75105 Uppsala, Sweden
email: stefane@it.uu.se, andreas.hellander@it.uu.se

[2] *Department of Computer Science, University of California–Santa Barbara,*
Santa Barbara, California 93106, USA.
email: bdrawert@cs.ucsb.edu

1 Introduction

Stochastic simulation methods are frequently used to study the behavior of cellular control systems modeled as continuous-time discrete-space Markov processes (CTMC). Compared to the most frequently used deterministic model, the reaction rate equations, the mesoscopic stochastic description can capture effects from intrinsic noise on the behavior of the networks [1, 8, 25, 26, 29].

In the discrete mesoscopic model the state of the system is the copy number of the different chemical species and the reactions are usually assumed to take place in a well-stirred reaction volume. The chemical master equation is the governing equation for the probability density, and for small to medium sized systems it can be solved by direct, deterministic methods [10, 11, 15, 21, 24, 28]. For larger models however, exact or approximate kinetic Monte Carlo methods [17, 18] are frequently used to generate realizations of the stochastic process. Many different hybrid and multiscale methods have also emerged that deal with the typical stiffness of biochemical reactions networks in different ways, see e.g. [2, 6, 19, 22, 23, 27].

Many processes inside the living cell can not be expected to be explained in a well-stirred context. The natural macroscopic model is the reaction-diffusion equation and it has the same limitations as the reaction rate equations. By discretizing the space with small subvolumes it is possible to model the reaction-diffusion process by a CTMC in the same formalism as for the well-stirred case. A diffusion event is now modeled as a first order reaction from a subvolume to an adjacent one and the state of the system is the number of molecules of each species in each subvolume. The corresponding master equation is called the reaction-diffusion master equation (RDME) and due to the very high dimensionality it cannot be solved by deterministic methods for realistic problem sizes.

The RDME has been used to study biochemical systems in [7, 14]. Here the next subvolume method (NSM) [7], an extension of Gibson and Bruck's next reaction method (NRM) [16], was suggested as an efficient method for realizing sample trajectories. An implementation on a structured Cartesian grid is freely available in the software MesoRD [20].

The method was extended to unstructured meshes in [12] by making connections to the finite element method (FEM). This has several advantages, but the most notable one is the ability to handle complicated geometries in a flexible way which is particularly important when internal structures of the cell must be taken into account.

This manual describes the software URDME which implements the unstructured extension of NSM as suggested in [12]. The purpose with the code is to provide an efficient, modular implementation that is easy to use for simulating and studying a particular model

*To whom correspondence should be addressed.

in an applied context, but also for developing and testing new approximate methods. We achieve this by relying on commercial software for the geometry definition, meshing, preprocessing and visualization and use a highly efficient computational core written in `Ansi C`. This keeps the implementation of the actual Monte Carlo simulation small and easily extendible, while the user benefits from the advanced pre- and postprocessing capabilities of modern FEM software. In URDME, we have chosen to provide an interface to `Comsol Multiphysics 3.5a` [3].

The rest of this manual is organized as follows. Section 2 summarizes the major changes to URDME. Section 3 describes the software requirements, installation procedure and testing of URDME,. An overview of the code structure is also offered in Section 4. The details concerning the input to the code, the provided interface to `Comsol Multiphysics 3.5a` and the way models should be specified are found in Section 5. Finally, a URDME model is set up and simulated in a step-by-step manner in Section 6. In appendix A we recapitulate the mesoscopic reaction-diffusion model and show how the stochastic diffusion intensities are obtained from a FEM discretization of the diffusion equation.

2 Summary of major changes

Below, we summarize the major changes compared to URDME 1.0[4].

- URDME 1.1 uses Comsol Multiphysics 3.5a to specify the part of the model related to the geometry. Comsol Multiphysics 3.4 is no longer supported.
- The Matlab interface has been substantially updated and model compilation/linking has been greatly simplified.
- Core solvers are now implemented as command line solvers for easier integration of contributed solvers and more flexibility in result generation work flows. The mex-interface from URDME 1.0 has been (temporarily) disabled.

3 Obtaining and installing URDME 1.1

3.1 System requirements and software dependencies

- Linux or Apple OSX operating system.
  (Untested, but may work under Cygwin or similar software)
- Matlab
  - 2008a, 2008b, 2009a, 2009b tested
  - Command line interface must be installed
- Comsol 3.5a (with appropriate patches)
  - Must have Matlab integration components installed
- GCC (Xcode on apple computers)
  - Executables `gcc` and `make` must be in the path.
  - Standard libraries must be installed

3.2 Installation procedure

1. Obtain latest release of URDME from sourceforge.net repository:
   http://sourceforge.net/projects/urdme
   Note: as the the writing of this document, the latest version of URDME is v1.1
   Download the file **urdme-1.1.tar.gz**

2. Unpack the archive.
   This can be done by running the command "**tar -zxvf urdme-1.1.tar.gz**" in a terminal. Often it is possible to double click the file icon in the operating system's graphical file manager.

3. Run the installation script with 'system administrator' privileges.
   In a terminal, change directory to the directory **urdme-1.1** created from the downloaded archive.
   run the script **install.sh** with "root" or system administrator privileges. This can usually be done by running the command "**sudo ./install.sh**". Follow the prompts of the installation script, the default values should be sufficient for most users. If the installation script runs with no errors, URDME should be installed correctly.

3.3 Testing of the installation *and* Quick start guide

1. Open Comsol Multiphysics
   Open model file. E.g. `urdme-1.1/examples/mincde/coli.mph`

2. From Comsol, start Matlab
   `File > Client/Server/MATLAB > Connect to MATLAB`

3. Initialize Matlab environment

   – Change Matlab "Current Folder" to the folder for the URDME model you wish to simulate.
     E.g. at the Matlab command prompt type **cd urdme-1.1/examples/mincde/**

4. Export model geometry from Comsol to Matlab

   – Update Model data.
     `Solve > Update Model`

   – Export data.
     `File > Export > FEM Structure as 'fem'`

5. Solve Model. At the Matlab command prompt type:
   `fem2 = urdme(fem,'fange')`
   or
   `fem2 = urdme(fem,@fange,{'Propensities','fange'})`

6. Visualize results. At the Matlab command prompt type:
   `postplot(fem2,'Tetdata','MinD_m')`

7. Revising the model.

   – `coli.mph` (Comsol model geometry, edited via the Comsol interface)
     Physical geometry and mesh properities
     Names of chemical species
     Diffusion coefficients

- `fange.m` (matlab pre-processing script)
  Stoichiometry matrix
  Dependency graph
  Initial conditions of chemical species
  Custom diffusion rules (i.e. support for membrane-only species)
- `fange.c`
  `Ansi C` functions defining the propensity (rate) of each reaction channel.

Repeat steps 4-6.

4 Code overview

In this section we give an overview of the structure of the code. The computational core of URDME is an efficient implementation of NSM and a small set of routines written in `Ansi C` or `Matlab`. Table 4.1 shows the directory structure of URDME together with a short description of each routine.

URDME consists of three logical layers. The top layer is an interface to an external finite element mesh generator and pre-/post-processing engine: `Comsol Multiphysics 3.5a`. The bottom layer is a set of highly efficient compiled `Ansi C` solvers, either the core NSM [7] solver or a contributed plugin solvers (e.g DFSP [5]). The middle layer is a set of functions implemented in the `Matlab` environment, designed to integrate the top and bottom layers and facilitate powerful data processing and visualization, as well as custom model development. Together these layers form a software package that enables the development and efficient simulation of complex and powerful models of spatial stochastic phenomena.

URDME uses an interface to `Comsol` to obtain geometry and finite element mesh information for the problem. The model geometry is created in `Comsol` and then assembled and the information is exported to `Matlab` via the `Comsol-Matlab` interface (see section 3.3 for procedure), usually as the variable `fem`. Once is imported, the user executes the `urdme()` command, and passing `fem` and the name of a model script file as parameters (and optionally solver parameters). Next `urdme()` uses the script `fem2rdme` assembles the matrix with diffusion rate constants D and the lumped mass matrix M into fields of the structure `fem.urdme`. The the model script file is then executed, with the `fem` structure passed in as a parameter. Next, through the function `urdme_compile()` the `Makefile`s are used to automatically compile a solver for this model, combing the specified solver core with the model specific propensity functions. When the compilation process is complete, the `fem.urdme` structure is serialized to a `.mat` file which is passed to the compiled solver for efficient simulation of the model. During the simulation, the solver indicates the progress back to the `Matlab` interface. When the simulation is complete, it returns the solution trajectory to the to the `Matlab` interface via another `.mat` file which is then imported.

The URDME model structure is designed for both efficiency and flexibility. A model is defined by three separate files, one used by each layer of URDME. The geometry of the model is defined in a `Comsol .mph` file, as well as the names and diffusion rates for each chemical species. The middle layer uses `Matlab` script model file defines the stoichiometric matrix, the dependency graph, and the initial state of the system. The stoichiometric matrix defines the effect of the chemical reactions on the state of the system. The dependency graph indicates the reaction rates that need to be updated after a given reaction event has occurred. The low level solvers use a reaction propensity file. The rates of the chemical reactions in the model are calculated by functions defined in an `Ansi C` file that is compiled into the solver core. Using compiled reaction rates allows for efficient simulation of the model by URDME.

Directory	File(s)	Description
bin	urdme_init	Environmental variable helper program.
build	Makefile	'make' input file for automatic solver compilation.
	Makefile.nsm	Makefile for NSM solver.
comsol	fem2rdme.m	`Matlab`-script converting `Comsol`'s FEM-struct to a valid `urdme`-input.
	rdme2fem.m	`Matlab`-script for conversion of the output of `urdme`to the solution format in `Comsol`. The purpose is to obtain a valid FEM-struct so that e.g. `Comsol`'s post-processing can be used.
doc	manual.pdf	The most recent version of this manual.
include	matmodel.h	Functions to serialize data to/from solvers.
	propensities.h	Definition of the propensity function datatype.
	report.h	Header for report.c.
msrc	urdme.m	
	urdme_startup.m	Initializes URDME.
	urdme_complie.m	Automatic solver compilation.
	urdme_validate.m	Input validation.
	rdme2mat.m	Serialize model data for input to solvers.
src	report.c	Report function used in `urdme`.
src/nsm	nsm.c	NSM solver main file.
	nsmcore.c	NSM solver computational core.
	nsm.h	Header for NSM solver.
	binheap.c	The binary heap used in NSM solver.
	binheap.h	Header for binheap.c.
examples	(various)	Contains files specifying the example studied in detail in Section 6.

Table 4.1: Overview of the files and routines that make up URDME.

5 Details and specifications

In this section we give a detailed description of the input to `urdme` and explain how the coupling between the Comsol/Matlab interface and the core solvers works.

5.1 Input to the core solver

Core solvers have two arguments: the path to an input file in *.mat* format and a name of an output file on which to store the trajectory that is generated. The input file will contain all the data structures the solver needs, each with its specific name. The `urdme` C-core contains utility routines to extract this data from the input file.

The main steps involved in launching a core-solver is oulined below, along with the routines that perform the different tasks. Generally, the user does not have to know or

perform all these steps manually: they are all wrapped in the main routine `urdme` in `urdme.m`. However, user's planning to write a plug-in solver for `urdme` will benefit from a more detailed knowledge of the code structure.

- *Process the `.mph` model file.* This is achieved by exporting the FEM-structure from the `Comsol` workspace to the `Matlab` workspace and invoking the routine `fem2rdme`. This will initialize a new structure, `fem.urdme` as a field in the original fem-structure. After calling `fem2rdme`, `fem.urdme` will contain the fields `D`, `vol`, `sd`, i.e. those data structures directly related to the geometry-part of the model and the unstructured mesh.
- The next step is typically to invoke the .m model file to initialize the remaining, essential data structures, `N`, `G`, `u0`, `tspan` and possibly `data`. They should all be added to the `fem.urdme` struct. Typically, the `.m` model files also modifies some of the data structures added by `fem2rdme` in the previous step.
- After all required field in the `fem.urdme` struct have been initialized, `urdme_validate` is used to perform some error checking on the input (it also checks for missing data).
- Next, all fields in `fem.urdme` is serialized to a `.mat` file: the input file using `rdme2mat`.
- After specifying the propensities in a ANSI C source file, the solver(s) are compiled using `urdme_compile` and then launched by a systems call from `Matlab`(or directly from the command line).
- The input file is now read by the core solver, using the utility routine `read_model`. `read_model` allocates, initializes and return a C-struct called `urdme_model`. These routines and typedefs are found in `matmodel.h`. A `urdme_model` struct is then the sole input to the routine `nsm` found in `nsm.c`. `nsm` unpacks the structure and calls the main simulation routine `nsm_core` found in `nsmcore.c`. A similar construction should be used by contributed solvers for easy integration, c.f. Section 7.
- After successful simulation, a result trajectory is written to an output file in .mat format. This file is the loaded in `Matlab` and added to the fem-struct by `urdme_addsol` for visualization using `Comsol` routines or custom `Matlab` scripts.

Table 5.1 summarizes the input to the core NSM solver. For precise type definitions, consult the preamble of the source file 'nsm_core.c'. While specific for the NSM solver, most of the input data are likely to be needed by any simulation routine/solver. Furthermore, contributed plug-in solvers should try to accept the same set of input as the NSM solver for full compatibility across models.

5.2 Specifying propensities for chemical reactions

The propensity functions are supplied to `urdme` as an `Ansi C` model file. The precise form of the propensity functions is defined by the data type `PropensityFun`.

`PropensityFun` is defined in the header 'propensities.h' (found in the 'include' directory) as

```
typedef double (*PropensityFun)(const int *x, double t, double vol,
                                const double *data, int sd);
```

The arguments to a `PropensityFun` are described in Table 5.1. Note that non of the current solvers are capable of simulating Markov chains with explicitly time-dependent propensities. The second argument, the current time, is included in the typedef at this stage only for completeness and for support for future plug-in solvers.

Below is a commented example of a model file defining a simple chemical system composed of a single species X undergoing a dimerization reaction.

Name	Type	Description
Ncells	scalar (int)	Number of subvolumes.
Mspecies	scalar (int)	Number of different species. This also defines Ndofs:= Mspecies×Ncells.
Mreactions	scalar (int)	Number of reactions.
M1	scalar (int)	Number of inline propensities.
dsize	scalar (int)	Size of the data vector used in the propensity function.
u0	Matrix (Mspecies×Ncells) (int)	u0(i, j) gives the initial number of species i in subvolume j.
tspan	vector (double)	An increasing sequence of points in time where the state of the system is to be returned.
prop	Vector[Mreactions-M1] (PropensityFun)	Propensity function pointers. See Section 5.2 for details.
report	ReportFun	Pointer to a report function. This function is called every time the chain reaches a value in tspan.
vol	Vector[Ncells] (double)	The volume of the macroelements, i.e. the diagonal elements of the lumped mass-matrix M.
sd	Vector[Ncells] (int)	The subdomain numbers of all subvolumes. See Section 6 for more details.
data	Matrix (dsize×Ncells) (double)	Generalized data vector. A pointer to column j is passed as an additional argument to the propensities in subvolume j.
D	Sparse matrix (Ndofs× Ndofs) (double)	The *transpose* of the diffusion matrix $M^{-1}K$ obtained from the FEM discretization of the macroscopic diffusion equation, cf. (A.5). Each column in D (i.e. each row in $M^{-1}K$) corresponds to a subvolume, and the non-zero coefficients $D(i, j)$ give the diffusion rate constant from subvolume i to subvolume j.
N	Sparse matrix (Mspecies× Mreactions) (int)	The stoichiometry matrix. Each column corresponds to a reaction, and execution of reaction j amounts to adding the jth column to the state vector.
G	Sparse matrix (Mreactions× [Mspecies+Mreactions]) (int)	Dependency graph. The first Mspecies columns correspond to diffusion events and the following Mreactions columns to reactions. A non-zeros entry in element i of column j indicates that propensity i needs to be recalculated if the event j occurs. See Section 6 for examples.

Table 5.1: Input arguments to `urdme`. For more details, see the source file 'nsmcore.c'. All data in the table will be passed to the core simulation routine via a C-struct `urdme_model` except `prop` and `report` that are specified in separate C files. For all sparse matrices, the compressed column sparse (CCS) format is used. This is the same format `Matlab` uses and online documentation is available.

```
/* Propensity definition of a simple dimerization reaction. */
#include <stdlib.h>
#include <stdio.h>
/* Type definition for propensity functions: */
#include "propensities.h"

/* Rate constant. */
const double k = 1.0e-3;

double rFun1(const int *x, double t, double vol, const double *data, int sd)
/*  X + X -> 0. */
{
  return k*x[0]*(x[0]-1)/vol;
}

PropensityFun *ALLOC_propensities(void)
/* Allocation. */
{
  PropensityFun *ptr = MALLOC(sizeof(PropensityFun));
  ptr[0] = rFun1;

  return ptr;
}

void FREE_propensities(PropensityFun *ptr)
/* Deallocation. */
{
  FREE(ptr);
}
```

In addition, any model file *must* implement the following routines:

- `PropensityFun *ALLOC_propensities(void)`
- `void FREE_propensities(PropensityFun *ptr)`

The first function should allocate and initialize an array of function pointers to the propensity functions and return a pointer to this array. This is the function that the core solvers will call to access the rate functions.

The second function should deallocate the pointer `ptr` but sometimes additional actions need to be implemented.

For further examples, see Section 6.

6 Example: Min oscillations in *E. Coli*

In this section we describe the general workflow involved in setting up and simulating a model in URDME using the `Comsol` and `Matlab` interfaces. The major steps involved are:

1. *Specify the model.* This involves (at least) to define the geometry, mesh, initial conditions and the chemical reactions in the model. In URDME, this will in most cases require the generation of three model files: a `Comsol` model file `model.mph`, a Matlab model file `model.m` and a reaction propensity C file `model.c`.

   In `Comsol Multiphysics 3.5a` a geometry can be created and a discretization of the diffusion equation with Neumann boundary conditions is readily obtained (this

will give the jump coefficients on the unstructured mesh). In `Matlab`, a model file that defines the stoichiometry matrix N and the dependency graph G can easily be specified. The chemical reactions will be specified in a model file written in ANSI C.

2. *Export the FEM structure from Comsol to the Matlab workspace.* After defining the geometry and meshing your model, export the FEM structure from Comsol to the Matlab workspace via the built-in Comsol/Matlab coupling.

3. *Run the simulation.* The core solver is launched from the Matlab workspace via the interface implemented in `urdme.m`. As input, you will have to specify the .m and .c model files. Internally, `urdme.m` uses `fem2rdme.m` to initialize a field called *urdme* in the FEM structure. The `model.m` file then appends data to this field.

4. *Postprocessing.* After a normal termination of the core solver, a trajectory of the stochastic process will be attached to the FEM structure. At this point, you can use all postprocessing options available in `Comsol`to visualize the results. If you have other needs not covered by the built-in routines, you can implement your own postprocessing routines in `Matlab`.

To illustrate the above steps in some more detail, we will reproduce simulations of the Min system from[14]. The geometry will be a model of an *E. coli* bacterium. It is rod shaped with length 3.5µm and diameter 0.5µm. The reactions and parameters of the model can be found in Table 6.1.

$$\text{MinD\_c\_atp} \xrightarrow{k_d} \text{MinD\_m} \qquad \text{MinD\_c\_atp} + \text{MinD\_m} \xrightarrow{k_{dD}} 2\text{MinD\_m}$$
$$\text{Min\_e+MinD\_m} \xrightarrow{k_{de}} \text{MinDE} \qquad \text{MinDE} \xrightarrow{k_e} \text{MinD\_c\_adp} + \text{Min\_e}$$
$$\text{MinD\_c\_adp} \xrightarrow{k_p} \text{MinD\_c\_atp}$$

Table 6.1: The chemical reactions of the MinD/MinE model. The constants take the values $k_d = 0.0125\mu m^{-1}s^{-1}$, $k_{dD} = 9 \times 10^6 M^{-1}s^{-1}$, $k_{de} = 5.56 \times 10^7 M^{-1}s^{-1}$, $k_e = 0.7s^{-1}$ and $k_p = 0.5s^{-1}$.

6.0.1 Setting up the model for simulation

Defining the geometry and diffusion rates in Comsol The following steps will show how to create the `Comsol` model file. If you don't want to go through all the steps yourself, open the example file `coli.mph` in the `examples/mincde` folder.

1. Open `Comsol` and select 'Chemical engineering module – Mass transport – Diffusion – Transient analysis' (3D). In the 'Dependent variables' field write `MinD_c_atp, MinD_m, Min_e, MinDE, MinD_c_adp`. These are the names of the variables that we will use. Select *Lagrange – Linear* elements and press 'OK'.

2. Next we create the geometry. We will build the rod shaped domain from two spheres and one cylinder. Press the 'Cylinder' button and in the radius and height field enter `0.5e-6` and `3.5e-6` and press 'OK'. You should now see a cylinder in your workspace. In the 'Draw mode' action bar, press 'Zoom extents' in order to get a better view of the domain. Press the 'sphere' button and enter `0.5e-6` in the radius field and press 'OK'. Create another identical sphere but enter `3.5e-6` as the z-coordinate. Select all three figures and press the 'union' button and then the 'Delete interior boundaries' button.

3. Having defined the species and the geometry, the next step is to specify the parameters in the model. In the menu 'Physics – Subdomain settings', choose subdomain 1 and

set the diffusion constants to 2.5e-12 for MinD_c_adp, MinD_c_atp and Min_e. For MinDE and MinD_m the diffusion constant should be 1e-14.

! *MinDE and MinD_m are membrane bound species, hence their lower diffusion constants. We have not specified this explicitely at this stage, but will do so later in the Matlab model file.*

4. In order to be able to distinguish between the interior of the bacterium and the membrane, we must also create two domains. One interior domain that represent the cytoplasm and one boundary domain that represent the membrane. This is done by defining rdme_sd as an expression with different value in the different subdomains. It can then be used to find the nodes on the boundary and in the interior. Select Options > Subdomain expressions and enter rdme_sd with value 1 and click 'OK'. Select Options > Boundary expressions and select all boundaries (there should be 12 of them). Enter rdme_sd with value 2. Finally select Options > Global expressions and enter rdme_sdlevel with value 2 indicating that the *lowest* dimension where rdme_sd is defined was on the surfaces.

5. In the Mesh menu, select Mesh > Free mesh parameters and choose Custom mesh size. Set the maximum element size to 1e-7 and press Initialize mesh. Now select Solve > Update model. Make sure that you are connected to Matlab, if not, connect via File > Client/Server/Matlab > Connect to MATLAB. Then export the FEM structure to the Matlab workspace from the File > export menu.

Specifying the chemical reactions The chemical reactions are specified in a separate model file in ANSI C. This file will be given as input to URDME, which will compile and launch a core solver. Every time the reaction propensity file is changed, the core solver needs to be recompiled, but this will be automatically detected by URDME. The way the reaction propensity functions are specified are explained in more detail in Section ??, which we recommend that you read before continuing with this example.

The following code specifies the reaction propensity model .c file for the reactions in Table 6.1. This file is located in examples/mincde/fange.c in the URDME installation directory. Either open that file, or create a new one of your own, entering the code below.

```
#include <stdlib.h>
#include "propensities.h"
#include "mex.h"

#define MinD_c_atp 0
#define MinD_m    1
#define MinD_e    2
#define MinDE    3
#define MinD_c_adp 4

#define CYTOSOL  1
#define MEMBRANE 2

#define NR  5

/* Rate constants. */

const double NA   = 6.022e23;
const double kd   = 1.25e-8;
const double kdd  = 9.0e6;
```

```
const double kde  = 5.56e7;
const double ke   = 0.7;
const double k_adp= 1.0;

/* Reaction propensities. */
double rFun1(const int *x, double t, double vol, const double *data, int sd)
/* MinD_c_atp -> MinD_m */
{
if (sd == MEMBRANE)
return kd*x[MinD_c_atp]/data[0];
return 0.0;
}

double rFun2(const int *x, double t, double vol, const double *data, int sd)
/* MinD_c_atp + MinD_m -> 2MinD_m */
{
return kdd*x[MinD_c_atp]*x[MinD_m]/(1000.0*NA*vol);
}

double rFun3(const int *x, double t, double vol, const double *data, int sd)
/* MinD_m + Min_e -> MinDE */
{
   return kde*x[MinD_m]*x[MinD_e]/(1000.0*NA*vol);
}

double rFun4(const int *x, double t, double vol, const double *data, int sd)
{
  return ke*x[MinDE];
}

double rFun5(const int *x, double t, double vol, const double *data, int sd)
/* MinD_c_adp -> MinD_c_atp */
{
   return k_adp*x[MinD_c_adp];
}


PropensityFun *ALLOC_propensities(void)
{
  PropensityFun *ptr = MALLOC(sizeof(PropensityFun)*NR);

  ptr[0]=rFun1;
  ptr[1]=rFun2;
  ptr[2]=rFun3;
  ptr[3]=rFun4;
  ptr[4]=rFun5;

  return ptr;
}

void FREE_propensities(PropensityFun* ptr)
{
  FREE(ptr);
```

```
}
```

There are a few points that deserves highlighting:

- Note the unit conversions given explicit in the bimolecular propensity function. The rate constants for the bimolecular reactions in this model are given in the unit $M^{-1}s^{-1}$ and need to be converted to mesoscopic rates. That is why we divide with Avogadros number times the volume of the subvolume. Also, the way we have set up the geometry model file, the volume is given in the unit m^3, and needs to be converted to L^3. URDME cannot keep track of matching the units between the different model files automatically: this is the modelers responsibility.
- Note how we use the input `sd` in the first reaction to make sure that it only occurs in subvolumes lying on the membrane. We have to make sure, however, that we keep track of what value we assigned to the different subdomains in the `Comsol` model file (the value of the expression `rdme_sd`).
- The input `t` passes the current time to the propensity function. This input is included in the typedef of the propensity function to make it more general and accommodate future needs. However, the core NSM-solver cannot deal with time dependent propensities. While an explicitly time dependent propensity might not generate any run-time errors, the resulting stochastic trajectory will not be a statisyically correct realization of the intended process.
- The first reaction in the model contains a scaling with the local length scale of the subvolume. For a uniform, Cartesian mesh this would simply have been the side lenghts of the cubes in the mesh, and could have been given by a constant. For the unstructured mesh however, this value will be different in every subvolume. It is readily obtained from `Comsol`, and is passed to the propensity function via the data vector `data`. `data` will be initialized with the correct values in the `Matlab` model file `fange.m`, described next.

Creating a .m model file Before we can run the simulation, we have yet to specify a few more data structures. We will also need to modify the diffusion rates that we obtain from the initial `Comsol`model so that the membrane-bound species only diffuse on the membrane. We have already prepared for this by labelling the subvolumes next to the boundary using the expression `rdme_sd` in the `Comsol` model file `coli.mph`.

Open the file `examples/mincde/fange.m`. We will walk through the contents of this file and explain what the different parts do. Additional information can also be found in the comments in the file.

1. *The stoichiometry matrix.* To execute the reactions, the solvers need to know the stoichiometry of the reactions. This is specified via a sparse matrix `N`. `N` has dimension $Mspecies \times Mreactions$. Entry (i, j) in N tells how species i changes upon execution of reaction j. The following lines of code will set up the stochiometry matrix for our example:

```
% Stoichiometry matrix. Every column corresponds to a reaction.
fem.urdme.N=sparse([-1  -1  0  0  1 ;...
                     1   1 -1  0  0 ;...
                     0   0 -1  1  0 ;...
                     0   0  1 -1  0 ;...
                     0   0  0  1 -1]);
```

2. *The dependency graph.* Efficient implementations of simulators for large systems uses a dependency graph to minimize the re-computation of rates. URDME requires that such a graph `G`, in the form of a sparse matrix, be submitted to the core NSM-solver. It should have dimensions $Mreactions \times (Mspecies + Mreactions)$. The following code sets up `G` for this example.

```
% Dependency graph. The first Mspecies columns tells which propensities
% needs to be updated that species diffuses. The following Mreactions
% columns does the same thing but for reaction events.
fem.urdme.G=sparse([1 0 0 0 0 1 1 0 0 1;...
                    1 1 0 0 0 1 1 1 0 1;...
                    0 1 1 0 0 1 1 1 1 0;...
                    0 0 0 1 0 0 0 1 1 0;...
                    0 0 0 0 1 0 0 0 1 1;]);
```

A non-zero entry at row i in column j means that propensity number i must be updated if species j diffuses ($j \leq Mspecies$) or if reaction j occurs ($j > Mspecies$).

! *A common reason to errors when developing a new model is errors in* `N` *or* `G`*.* A quick way of setting up the dependency graph is `fem.urdme.G = sparse(ones(Mreactions, Mspecies+Mreactions))`. This will make all propensities be recomputed after each event. While making the code run slower, this is guaranteed to be correct and can be useful when debugging your model file.

3. *The initial condition.* The initial condition can be set in a various of ways. Here, we want 4002 $MinD\_c\_atp$ and 1040 $MinE$ molecules to be randomly distributed in the entire bacterium.

```
% Specify the total number of molecules of the species.
nMinD = 4002;
nMinE = 1040;

u0 = zeros(Mspecies,Ncells);

% Random distribution on the voxels specified in the vector ''all''.
all = 1:Ncells;

ind = floor(NCells*rand(1,nMinD))+1;
for i=1:nMinD
   u0(5,all(ind(i))) = u0(5,all(ind(i)))+1;
end

ind = floor(Ncells*rand(1,nMinE))+1;
for i=1:nMinE
   u0(3,all(ind(i))) = u0(3,all(ind(i)))+1;
end

fem.urdme.u0 = u0;
```

We simply create a vector with the same number of random indices as the total number of molecules we want to distribute and then place them in their corresponding place in `u0`.

4. *Specifying the times to output the state of the system.* URDME will look for a vector `tspan` to determine when to output the state of the trajectory (the number of events generated in a typical realization often exceeds 10^9 so we can't output after each event). Here, we want to sample the system on the time interval $[0, 200s]$, with output each second. This is achieved by:

```
fem.urdme.tspan = 0:200.
```

5. *Membrane diffusion.* In order to make $MinD\_m$ and $MinDE$ diffuse only on the membrane, we will zero out all elements in the diffusion matrix that are in the cytosol. To obtain indices of those subvolumes we use the information in the *subdomain vector* `sd`. `sd` will be generated by the urdme interface upon calling the solver interface, and will contain the information encoded in the expression `rdme_sd` in the `Comsol` model file. For more details, see Section 5.

```
pm  = find(fem.urdme.sd == 2);
cyt = find(fem.urdme.sd == 1);
```

Remember that we gave `rdme_sd` the value 2 on the membrane and 1 in the interior. The diffusion matrix `D` will contain the rate constants for the diffusive events on the unstructured mesh. `D` is also generated from the `Comsol` model file when calling the solver, and will be available to the .m model file in `fem.urdme.D`. To (efficiently) zero out the correct entries in `D`, we first decompose the sparse matrix, find the entries using `pm` and `cyt` above, and then reassemble the matrix again (compensating for the removed entries by adjusting the diagonal of the matrix). All in all, the code to do this can be as follows:

```
D = fem.urdme.D';

% Decompose the sparse matrix.
[i,j,s] = find(D);

% And set all elements in the diffusion matrix corresponding to
% to the cytosol to zero.

ixremove = [find(ismember(i,ixremove)); find(ismember(j,ixremove))];
i(ixremove) = [];
j(ixremove) = [];
s(ixremove) = [];

% Reassemble the sparse matrix and adjust the diagonal entries.
ixkeep = find(s > 0);
D = sparse(i(ixkeep),j(ixkeep),s(ixkeep),Ndofs,Ndofs);
d = full(sum(D,2));
D = D+sparse(1:Ndofs,1:Ndofs,-d);

fem.urdme.D = D';
```

! *It is of fundamental importance that the columns of `D` sum to zero, and that all off-diagonal entries are positive. For an introduction to how `D` is constructed, see Appendix A. For a detailed account, consult [12].*

! *The way we have modeled membrane diffusion is simply by saying that the subvolumes closest to the membrane constitute the membrane layer. As the mesh become finer*

near the boundary, the thickness of this layer will decrease, eventually approaching a 2D model on the membrane. One can think of other ways of modeling the membrane diffusion. The most obvious is to explicitly draw the membrane as a separate (true) subdomain with a fixed thickness in the `Comsol` model file. This would usually mean that more subvolumes are needed to resolve that thin layer.

6. *The generalized data vector.* Finally, we need to set `fem.urdme.data` to contain the values of the length parameter for the subvolumes (it is needed in the first reaction, rFun1). To do this, we use the built-in `Comsol` function `postinterp`. `postinterp` can be used to evaluate any user-defined or pre-defined expression in any point in the domain. Here, we get the subvolume sizes by using the pre-defined expression h, evaluated in the vertices in the mesh, given in `dofs.nodes`.

```
% "postinterp" is a built-in Comsol function that evaluates any (valid)
% expression at a set of specified points. The predefined
% expression 'h' can be used to obtain the local length of each subvolume.

dofs = xmeshinfo(fem,'Out','dofs');
fem.urdme.data = postinterp(fem,'h',dofs.coords(:,1:Mspecies:end));
```

! *The vertices in the mesh can be found in several places in the FEM structure, e.g. in `fem.mesh.p`. However, the ordering of the degrees of freedom are not necessarily the same as the ordering of the mesh vertices. It is the dof-ordering that is used internally in the core solver, and thus we need to make sure that we pass the subvolume size in the correct order. Another way of achiving the same thing as above is:*

```
dofs = xmeshinfo(fem,'Out','dofs');
data = postinterp(fem,'h',fem.mesh.p);
fem.urdme.data = fem.urdme.data(dofs.nodes(1:Mspecies:end));
```

For more details concerning the internal ordering of the dofs, consult the `Comsol` user's manual. The interface routines `fem2rdme` and `rdme2fem` also contain useful information on this matter.

6.0.2 Running the simulation

With all three model files set up correctly, we are now ready to launch the simulation. In `Matlab`, change the current working directory to `examples/mincde` (or if you have prepared your files in another directory, to that one). Initialize URDME by calling

```
>> urdme_startup;
```

Next, to launch the simulation, call the main interface routing in `urdme.m`

```
>> fem = urdme(fem,@fange,{'Propensities','fange'});
```

URDME will now extract information from the `Comsol` model file, the `fange.m` model file and compile the core solver, linking to the propensities specified in `fange.c`, and the call the solver by making a systems call.

6.0.3 Postprocessing

If the simulation in the previous step completed without errors, the model structure will now contain a realization of the stochastic process. To visualize the trajectory, we can use any visualization routine `Comsol` provides, or create routines of our own. First, to look at the `MinD_m` distribution on the membrane at the final time we can use the routine `postplot`:

```
>> postplot(fem,'Tetdata','MinD_m');
```

To visualize the result at any other time, e.g after 300s:

```
>> postplot(fem,'Tetdata','MinD_m','T',300);
```

! *You can specify any time in the time interval you simulated, but if you give a time point that lies between two point in* `tspan` `Comsol` *will do interpolation to approximate the result at that time point. While this is a sensible approach if the solution varies smoothly such as in the corresponding deterministic PDE model, generally it makes little sense for a realization of the stochastic process.*
To visualize a species inside the domain, we can do as follows:

```
>> postplot(fem,'Slicedata','MinD_c_atp');
```

There are many more options that can be passed to `postplot` to control the plot produced. For a detailed account , see the `Comsol` documentation, or, if you are an experienced `Matlab` user:

```
>> help postplot;
```

If you prefer to work within the `Comsol` GUI for visualization, you can import the FEM structured with the attached stochastic trajectory back into `Comsol`. We have added a field to the structure, `fem.urdme`, and `Comsol` does not recognize this data. Before importing, we need to make a copy of our structure where we have removed the URDME specific data. This can be easily achieved by:

```
>> fem2 = rmfield(fem,'urdme');
```

Then, from the `Comsol`GUI, import the new structure (fem2): 'File > Import > FEM structure...'. You can now visualize the trajectory using the menu 'Postprocessing > Plot Parameters'.

7 Integrating solvers with URDME

In this section we will describe how to integrate a third party spatial stochastic solver into URDME using the DFSP [5] plugin as an example. Table 7.1 describes the files that make up the DFSP plugin. We recommended that plugin developers follow the the format of this plugin when integrating their own solvers.

URDME Plugins have three main components: the solver executable, the makefile, and (optionally) a pre-execution script. Each part is described in detail below.

When the middle level interface calls the solver executable, it passes all model and geometry data to the solver via a `.mat` data file. The names of the input file and the output file the solver saves the solution trajectory to are both specified as command line arguments (i.e. in the `argv` parameter to the `main()` function). The core URDME distribution includes routines to read and parse this data file into a C-style struct. The solver is then called with the model struct as a parameter. Once the solver has finished simulating the model, it attaches the calculated solution trajectory to model structure. The solution trajectory is then serialized to the output file using supplied routines. When the solver has completed its

Directory	File(s)	Description
build	Makefile.dfsp	Solver makefile, for building model solvers automatically when calling `urdme`. The name of this file is very important: the automatic compilation process looks for a makefile that is suffixed with the name of the solver (in lower case). This makefile compiles the solver with the model's propensity functions into the low-level executable which is the called by the middle-level interface.
src/dfsp	dfsp.c	Solver entry point and data initialization code. Contains `main()` and data structure setup routines. The initialization procedure takes the `.mat` file (which is a serialization of the `fem.urdme` structure from the Matlab interface) and instantiates a `urdme_model` struct (defined in "include/matmodel.h").
	dfsp.h	Header file containing all the function prototypes necessary for DFSP
	dfspcore.c	Main entry point for solver execution: `dfsp_core()` function.
	dfsp_reactions.c	Helper functions to process reaction events.
	dfsp_diffusion.c	Helper functions to process diffusion events.
msrc	urdme_init_dfsp.m	Pre-execution script to initialize data structures before solver is called. When executing a model with a specified solver (hereafter SOLVER), the `urdme` interface looks for a Matlab function named `urdme_init_SOLVER()` that is defined in the file "urdme_init_SOLVER.m"

Table 7.1: Overview of the files that make up the DFSP plugin to URDME.

execution, the middle level interface imports the serialized solution trajectory, validates the data and makes it available to the post-processing and visualization routines. The logical separation of solvers from the rest of the URDME software enables streamlined development and debugging of new computational methods.

For efficient simulation of URDME models, it is necessary to compile the model specific propensity functions with the routines of the solver chosen to perform the simulation. The solver specific makefiles are responsible for this compilation. For illustration we will use the DFSP plugin and the "mincde" `fange` model as a example (i.e. replace 'dfsp' with the name of solver you wish to integrate). From the middle-level Matlab interface, when the `urdme()` function is call with the parameter {`'Solver','dfsp'`}, URDME attempts to compile the executable "./urdme/fange.dfsp" from the propensity function file "fange.c" and solver files using the compilation specified in "Makefile.dfsp" in the "build" subdirectory of URDME. The makefile is responsible for all necessary steps of the compilation process and the target executable is built in the ".urdme" subdirectory of the current working directly, and is named according to the propensity file ("fange.c") and solver (*dfsp*), thus "./urdme/fange.dfsp". The automatic compilation process is designed for ease of use from the middle-level Matlab interface.

Often spatial stochastic simulation methods require additional processing of geometry and model data before execution can proceed. In URDME, this is accomplished through the

use of a specifically named Matlab function found in the *msrc* subdirectory of the URDME distribution. For the DFSP plugin, this file is named "urdme_init_dfsp.m". The function defined in this file must take as parameters the *fem* data structure and a variable number of additional arguments (i.e. `varargin`). If `urdme()` is called with solver arguments, that cell-array is passed as the second argument to this function. Implementation of a pre-processing script provides method developers with a powerful and flexible way to perform any necessary data transformations for their specific solvers.

Acknowledgment

The authors are grateful to Per Lötstedt and Linda Petzold for valuable input during the development of URDME.

Funding: The Swedish Graduate School in Mathematics and Computing (AH, SE), The Royal Swedish Academy of Sciences scholarship FOA08H-109, FOA09H-63, FOA09H-64 (AH), SSF A3 02:124 (AH, JC), U.S. NIH grant R01EB7511, U.S DOE award DE-FG02-04ER25621, U.S. NSF IGERT DGE-02-21715, Institute for Collaborative Biotechnologies Grant DAAD19-03-D-0004 (BD).

References

[1] Naama Barkai and Stanislav Leibler. Circadian clocks limited my noise. *Nature*, 403:267–268, 2000.

[2] Yang Cao, Dan T. Gillespie, and Linda Petzold. Multiscale stochastic simulation algorithm with partial equilibrium assumption for chemically reacting systems. *J. Comput. Phys.*, 206:395–411, 2005.

[3] Comsol Inc., Stockholm, Sweden. *Comsol Multiphysics Reference Guide Version 3.4*, 2008. `http://www.comsol.com`.

[4] Josef Cullhed, Stefan Engblom, and Andreas Hellander. The URDME manual version 1.0. Technical Report 2008-022, Department of Information Technology, Uppsala University, 2008.

[5] Brian Drawert, Michael J. Lawson, Linda Petzold, and Mustafa Khammash. The diffusive finite state projection algorithm for efficient simulation of the stochastic reaction-diffusion master equation. *J. Chem. Phys.*, 132(7):074101, 2010.

[6] Weinan E, Di Liu, and Eric Vanden-Eijnden. Nested stochastic simulation algorithm for chemical kinetic systems with disparate rates. *J. Chem. Phys.*, 123, 194107, 2005.

[7] Johan Elf and Måns Ehrenberg. Spontaneous separation of bi–stable biochemical systems into spatial domains of opposite phases. *Syst. Biol.*, 1(2), 2004.

[8] Michael B. Elowitz, Arnold J. Levine, Eric D. Siggia, and Peter S. Swain. Stochastic gene expression in a single cell. *Science*, 297(5584):1183–1186, 2002.

[9] Stefan Engblom. *Numerical Solution Methods in Stochastic Chemical Kinetics.* PhD thesis, Uppsala University, 2008.

[10] Stefan Engblom. Spectral approximation of solutions to the chemical master equation. *J. Comput. Appl. Math.*, 2008 *(to appear)*.

[11] Stefan Engblom. Galerkin spectral method applied to the chemical master equation. *Commun. Comput. Phys.*, 5(5):871–896, 2009 *(to appear)*.

[12] Stefan Engblom, Lars Ferm, Andreas Hellander, and Per Lötstedt. Simulation of stochastic reaction–diffusion processes on unstructured meshes. *SIAM J. Scientific. Comp.*, 2008 *(to appear)*.

[13] Stewart N. Ethier and Thomas G. Kurtz. *Markov Processes: Characterization and Convergence.* Wiley series in Probability and Mathematical Statistics. John Wiley & Sons, New York, 1986.

[14] David Fange and Johan Elf. Noise-induced Min phenotypes in *E. coli*. *PLOSB*, 2(6):0637–0647, 2006.

[15] Lars Ferm and Per Lötstedt. Adaptive solution of the master equation in low dimensions. *Appl. Numer. Math.*, 59(1):265–284, 2009.

[16] Michael A. Gibson and Jehoshua Bruck. Efficient exact stochastic simulation of chemical systems with many species and many channels. *J. Phys. Chem.*, 104:1876–1889, 2000.

[17] Daniel T. Gillespie. A general method for numerically simulating the stochastic time evolution of coupled chemical reacting systems. *J. Comput. Phys.*, 22:403–434, 1976.

[18] Daniel T. Gillespie. Approximate accelerated stochastic simulation of chemically reacting systems. *J. Chem. Phys.*, 115(4):1716–1733, 2001.

[19] Eric L. Haseltine and James B. Rawlings. Approximate simulation of coupled fast and slow reactions for stochastic chemical kinetics. *J. Chem. Phys.*, 117(15):6959–6969, 2002.

[20] Johan Hattne, David Fange, and Johan Elf. Stochastic reaction–diffusion simulation with MesoRD. *Bioinformatics*, 21(12):2923–2924, 2005.

[21] Markus Hegland, Conrad Burden, Lucia Santoso, Shev MacNamara, and Hilary Booth. A solver for the stochastic master equation applied to gene regulatory networks. *J. Comput. Appl. Math.*, 205(2):708–724, 2007.

[22] Andreas Hellander. Numerical simulation of well stirred biochemical reaction networks governed by the master equation. *Licentiate thesis, Department of Information Technology, Uppsala University*, 2008.

[23] Andreas Hellander and Per Lötstedt. Hybrid method for the chemical master equation. *J. Comput. Phys.*, 227(1):127–151, 2008.

[24] Shev F. MacNamara. *Krylov and Finite State Projection Methods for Simulating Stochastic Biochemical Kinetics via the Chemical Master Equation.* PhD thesis, The University of Queensland, Australia, 2008.

[25] Harley H. McAdams and Adam Arkin. It's a noisy business! Genetic regulation at the nanomolar scale. *Trends in Genetics*, 15(2):65–69, 1999.

[26] Johan Paulsson, Otto G. Berg, and Mans Ehrenberg. Stochastic focusing: Fluctuation-enhanced sensitivity of intracellular regulation. *Proc. Nat. Acad. Sci. USA*, 97(13):7148–7153, 2000.

[27] Christopher V. Rao and Adam P. Arkin. Stochastic chemical kinetics and the quasi-steady-state assumption: Application to the Gillespie algorithm. *J. Chem. Phys.*, 118(11):4999–5010, 2003.

[28] Paul Sjöberg. *Numerical Methods for Stochastic Modeling of Genes and Proteins.* PhD thesis, Uppsala University, 2007.

[29] Mukund Thattai and Alexander van Oudenaarden. Intrinsic noise in gene regulatory networks. *Proc. Nat. Acad. Sci. USA*, 98:8614–8619, 2001.

A Stochastic chemical kinetics

In this section we briefly describe how reaction and diffusion events are modeled and how we obtain the diffusion rate constants when the domain is discretized using an unstructured mesh. For a fuller introduction to the subject along with many additional references, consult [9].

The computational core of URDME is based on the next subvolume method (NSM) [7], but it has been adapted to simulation on unstructured meshes by supporting a more general input format. Details concerning the actual simulation algorithms can be found in Appendix A.

A.1 Mesoscopic chemical kinetics

In a well-stirred chemical environment reactions are understood as transitions between the states of the integer-valued state space counting the number of molecules of each of D different species. The intensity of a transition is described by a *reaction propensity* defining the transition probability per unit of time for moving from the state x to $x - N_r$;

$$x \xrightarrow{\omega_r(x)} x - N_r, \tag{A.1}$$

where $N_r \in \mathbf{Z}^D$ is the transition step and is the rth column in the *stoichiometric matrix* N. Eq. (A.1) defines a continuous-time Markov chain over the positive D-dimensional integer lattice.

When the reactions take place in a container of volume Ω, it is sometimes useful to know that the propensities often satisfy the simple scaling law

$$\omega_r(x) = \Omega u_r(x/\Omega) \tag{A.2}$$

for some function u_r which does not involve Ω. Intensities of this form are called *density dependent* and arise naturally in a variety of situations [13, Ch. 11].

A.2 Mesoscopic diffusion

In the mesoscale model, a diffusion event is modeled as a first order reaction taking species S_l in subvolume ζ_i from its present subvolume to an adjacent subvolume ζ_j,

$$S_{li} \xrightarrow{a_{ij}\mathbf{x}_{li}} S_{lj}, \tag{A.3}$$

where $\mathbf{x}_{li}$ is the number of molecules of species l in subvolume i. On a uniform Cartesian mesh such as those used in MesoRD [20], the rate constant takes the value $a_{ij} = \gamma/h^2$ where h is the side length of the subvolumes and γ is the diffusion constant. In URDME we use an unstructured mesh made up of tetrahedra and the rate constants are taken such that the expected value of the number of molecules divided by the volume (the concentration) converges to the solution obtained from a consistent FEM discretization of the diffusion equation

$$u_t = \gamma \Delta u. \tag{A.4}$$

Using piecewise linear Lagrange elements and mass lumping, we obtain the discrete problem

$$u_t = M^{-1} K u \tag{A.5}$$

where M is the lumped mass matrix and $K = \{k_{ij}\}$ is the stiffness matrix. The rate constants on the unstructured mesh are then given by

$$a_{ij} = \frac{1}{\Omega_i} k_{ij}, \tag{A.6}$$

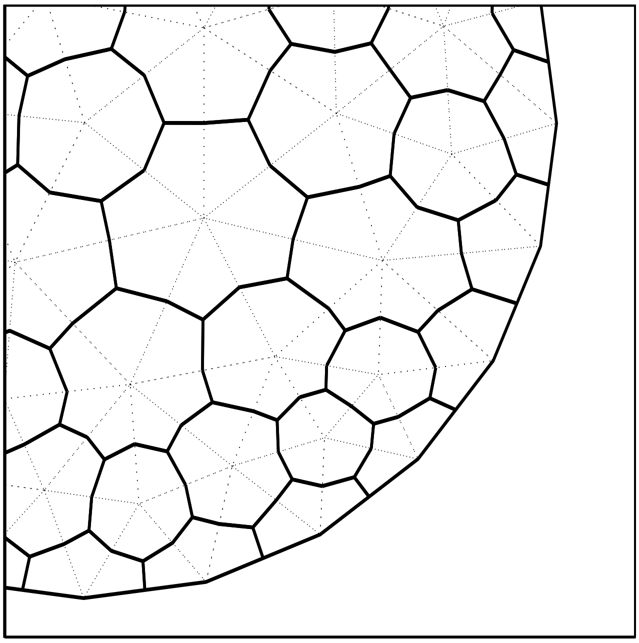

Figure A.1: A 2D example of an unstructured triangular mesh. The primal mesh is shown in dashed and the dual in solid. Within each dual element the system is assumed to be well-stirred, and molecules can jump from each dual cell to the neighboring ones.

where Ω_i is the diagonal entry of M and can be interpreted as the volume of the dual element associated with mesh node i (see Figure A.1). For more details, consult [12].

The assumption made in the mesoscopic model is that molecules are well-stirred within a dual cell. These dual cells correspond to the cubes of the staggered grid in a Cartesian mesh.

B Algorithms

One of the most popular algorithms to generate realizations of the CTMC in the well-stirred case is Gillespie's direct method (DM) [17]. Several algorithmic improvements of this method exist, one of them being the next reaction method (NRM) due to Gibson and Bruck [16].

The underlying algorithm in URDME is the next subvolume method (NSM) [7]. The NSM essentially combines ideas from NRM and DM in order to tailor the algorithm to reaction-diffusion processes.

For reference, we first state below both DM and NRM and then outline NSM.

Algorithm 1 Gillespie's direct method (DM)

Initialize: Set the initial state $\mathbf{x}$ and compute all propensities $\omega_r(\mathbf{x}), r = 1, \ldots, M_{\text{reactions}}$. Also set $t = 0$.

while $t < T$ **do**

Compute the sum λ of all the propensities.

Sample the next reaction time (by inversion), $\tau = -\log(\text{rand})/\lambda$. Here and in what follows, 'rand' conveniently denotes a uniformly distributed random number in $(0, 1)$ which is different for each occurrence.

Sample the next reaction event (by inversion); find n such that $\sum_{j=1}^{n-1} \omega_j(\mathbf{x}) < \lambda \,\text{rand} \le \sum_{j=1}^{n} \omega_j(\mathbf{x})$

Update the state vector, $\mathbf{x} = \mathbf{x} - N_n$ and set $t = t + \tau$.

end while

Algorithm 2 Gibson and Bruck's next reaction method (NRM)

Initialize: Set $t = 0$ and assign the initial number of molecules. Generate the dependency graph G. Compute the propensities $\omega_r(\mathbf{x})$ and generate the corresponding *absolute* waiting times τ_r for all reactions r. Store those values in a heap H.

while $t < T$ **do**

Remove the smallest time $\tau_n = H_0$ from the top of H, execute the nth reaction $\mathbf{x} := \mathbf{x} - N_n$ and set $t := \tau_n$.

for all edges $n \to j$ in G **do**

if $j \neq n$ **then**

Recompute the propensity ω_j and update the corresponding waiting time according to

$$\tau_j^{\text{new}} = t + \left(\tau_j^{\text{old}} - t\right) \frac{\omega_j^{\text{old}}}{\omega_j^{\text{new}}}.$$

else $\{j = n\}$

Recompute the propensity ω_n and generate a new absolute time τ_n^{new}. Adjust the contents of H by replacing the old value of τ_n with the new one.

end if

end for

end while

Algorithm 3 The next subvolume method (NSM)

Initialize: Compute the sum σ_i^r of all reaction rates ω_{ri} and the sum σ_i^d of all diffusion rates $a_{ij}\mathbf{x}_{si}$ in all subvolumes $i = 1, \ldots, N_{\text{cells}}$. Compute the time until the next event in each subvolume, $\tau_i = -\log(\text{rand})/(\sigma_i^r + \sigma_i^d)$, and store all times in a heap H.

while $t < T$ **do**

  Select the next subvolume ζ_n where an event takes place by extracting the minimum τ_n from the top of H.

  Set $t = \tau_n$.

  Determine if the event in ζ_n is a reaction or a diffusion event. Let it be a reaction if $(\sigma_n^r + \sigma_n^d)\,\text{rand} < \sigma_n^r$, otherwise it is a diffusion event.

  if Reaction event **then**

    Determine the reaction channel that fires. This is done by inversion of the distribution for the next reaction given τ_n in the same manner as in Gillespie's direct method in Algorithm 1.

    Update the state matrix using the (sparse) stoichiometry matrix N.

    Update σ_n^r and σ_n^d using the dependency graph G to recalculate only affected reaction- and diffusion rates.

  else {Diffusion event}

    Determine which species S_{ln} diffuses and subsequently, determine to which neighboring subvolume $\zeta_{n'}$. This is again done by inversion using a linear search in the corresponding column of D.

    Update the state: $S_{nl} = S_{nl} - 1$, $S_{n'l} = S_{n'l} + 1$.

    Update the reaction- and diffusion rates of subvolumes ζ_n and $\zeta_{n'}$ using G.

  end if

  Compute a new waiting time τ_n by drawing a new random number and add it to the heap H.

end while
