# Supplementary material for: URDME: a modular framework for stochastic simulation of reaction-transport processes in complex geometries
Source: BMC Syst Biol. 2012 Jun 22;6:76. doi: 10.1186/1752-0509-6-76 (PMC3439286; doi:10.1186/1752-0509-6-76)
Supplement: Additional file 6 — validation.pdf. Simulation results for a simple diffusion problem on the surface of a sphere and for the Min system [8,34,55,56,59,60]. [file 1752-0509-6-76-S6.pdf]

A Comparison of Simulation Accuracy with URDME, MesoRD and STEPS

an addendum to

URDME: a modular framework for stochastic simulation of reaction-transport processes in complex geometries

by

Brian Drawert, Stefan Engblom, Andreas Hellander

June 11, 2012

Abstract

This appendix shows simulation results using URDME and MesoRD for a simple diffusion problem. This problem illustrates the advantage of unstructured meshes: the ability to resolve processes on curved surfaces without causing a unnecessarily fine discretization. We also evaluate the accuracy of the software on a non-trivial reaction-diffusion system by simulating the example of Min oscillations presented in the main text of URDME, MesoRD and STEPS. The output metric used to study the results of the simulations is the mean oscillation period.

Simple diffusion to a target on the surface of a sphere

A major strength of using a tetrahedral and triangular mesh over a Cartesian mesh is the possibility to better resolve curved boundaries, and the ability to model 2D processes on membranes embedded in a 3D volume by diffusion on the surface mesh. To illustrate this, we used URDME and MesoRD to simulate diffusion on the surface of a sphere. When molecules hit a small circular patch on one of the poles of the sphere, they become absorbed. For a sphere with radius $1\mu m$, a diffusion constant $\gamma = 10^{-12}\ m^2$ and a patch with a small radius r, the exact solution is well approximated by [59, 60]

$$\tau = \frac{2 \ln \frac{2}{(1-\cos r)}}{(1+\cos r)} - 1$$

We used $r = 0.1$, giving $\tau \approx 5.01\ s$. Figure 1 shows simulation results for URDME for varying number of voxels. Diffusion in URDME is modeled using Comsol's capability to discretize diffusion on a curved surface. For details, consult the model file in Additional File 7. Figure 1 shows the results from simulations with URDME for varying mesh resolutions. As can be seen, even for the coarsest mesh with only 500 voxels, the error is $\approx 4\%$. For the finest mesh with 4343 voxels the error is as small as 0.2%.

In order to simulate this model in MesoRD, we created a corresponding 3D geometry consisting of the difference between two spheres, one with radius $1.05\mu m$ and one with radius $0.95\mu m$ for a membrane width of $100nm$. To model the target, we let a sphere with radius $0.1\mu m$ centered

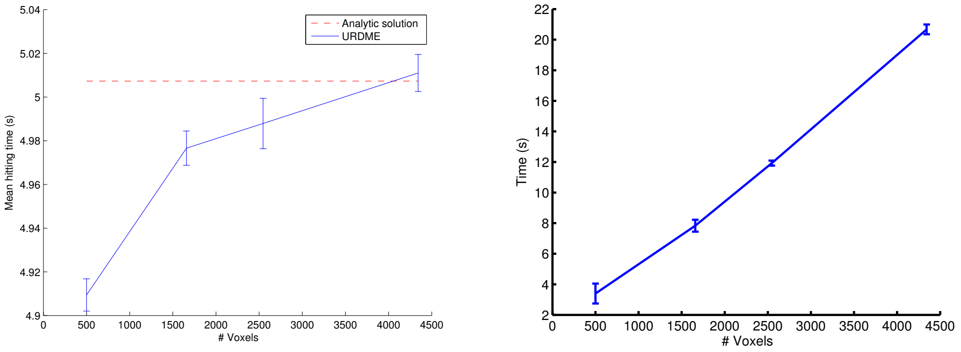

(a) (b)

Figure 1: **(a)** Mean hitting time for diffusion to a small target on the surface of a sphere as a function of the mesh resolution. **(b)** Simulation time to compute an estimate based on 10^4 diffusing particles . The error bars indicate a 95% confidence interval.

at $(x = 0, y = 1.0\mu m, z = 0)$ cut out a "lid" in the membrane volume. We also created a corresponding file with a membrane width of $w = 50nm$. Table summarizes results for simulations for a number of different voxel sizes q.

	Width 100 nm		Width 50 nm	
q (nm)	hitting time (s)	sim time (s)	hitting time (s)	sim time (s)
100	12.2	92	–	
50	7.1	597	*	
30	6.2	1460	7.2	1766
20	5.7	6132	6.3	3152
15	5.7	9118	6.0	6431
10	†		5.6	21056

Table 1: MesoRD simulations of mean hitting times of diffusion to a small target on a sphere for different membrane widths and different mesh resolutions. The analytical solution for a purely 2D surface is $\tau \approx 5s$. Dashes in the table indicates that the experiment was not conducted since the voxel size is larger than the width of the membrane. The * indicates that the experiment failed. For some reason, the molecules did not decay away as expected, instead the total number of molecules remained constant after a initial period of some decay. A possible explanation for this behavior is a disconnected membrane volume. The † indicates that the experiment was not conducted. In this case, the mesh consisted of 1.25×10^6 voxels and the simulation was deemed unfeasible. Also, the result from the two previous mesh sizes indicates that the hitting time converges to ≈ 5.7 for this membrane thickness.

The MinCDE system

The previous test was designed to illustrate and isolate the properties of the diffusion modeling. A more realistic and biologically interesting model is that of Min oscillations, presented as example 1 in the main paper and the model used to benchmark URDME 1.1.2, STEPS 1.3.0 and MesoRD 1.0 in the discussion section in the main paper. Here we present the results of the simulations

for the three different software packages. Accuracy is here measured based on the period of the oscillations. The mean period is computed from a time series constructed by computing the sum of the membrane bound MinD protein in one half of the bacterium. A simple power spectrum is then computed using the Fourier transform, and the period is taken to be the inverse of the frequency with maximal power. The temporal average profile is computed by slicing the bacterium in 20 slices perpendicular to the long-axis of the bacterium, and computing the mean copy number over those volumes and over time. The temporal average will have a minimum near the center of the cell, and maximum at the poles.

The URDME version of the model is an example model in our software distribution. It is an implementation of the Min model proposed in [8]. This model was originally developed with early versions of MesoRD, and the MesoRD version of the model was adopted from the SBML file appended as supplementary material to [8], modified to support the latest SBML format in MesoRD 1.0. The membrane in the MesoRD model is modeled as a $50nm$ thick volume surrounding the cytsol compartment. For the STEPS implementation of the model we exported the same tetrahedral mesh from Comsol that were used in the URDME runs into a STEPS format (the conversion script can be found in Additional File 7). Then we created a STEPS python script file that imports the mesh, sets up the model and executes the simulation. We include all the model files used for the comparison as part of Additional File 7. We would like to note that implementation of the model in STEPS was incomplete in the sense that the example model in our distribution includes diffusion of the membrane bound proteins on the surface of the bacterium, while STEPS does not yet support diffusion along surfaces. We could have modeled diffusion in the membrane in the same way as in the MesoRD model (as a 3D volume with a finite but small extension). However, we feel that this in counterintuitive to the approach of using unstructured meshes. Instead, we ran URDME and MesoRD with membrane diffusion constant zero for a direct comparison to STEPS.

	# voxels	No membrane diffusion period (s)	Membrane diffusion period (s)
URDME 1.1	1555	28.8	29.1
	2818	28.8	28.8
MesoRD 1.0	3076	28.8	31.3
	32000	30.0	31.3
STEPS 1.3	1301	28.8	–
	2149	28.8	–

Table 2: Mean oscillation period of MinD_m calculated from a time series with approximately 30 periods. The model with surface diffusion was not implemented in STEPS.

Table 2 shows the mean periods for different mesh resolutions. URDME and STEPS give the same period length, while MesoRD gives a slightly longer period. No analytical solution is available for this model, so it is not possible to say which software is more accurate, but all software packages produce similar results. The small difference could potentially be explained by a subtle difference in the models. In one of the reactions, MinD binds to the plasma membrane. This reaction involves a length scale parameter, and how to correctly choose this parameter for a general, curved surface is not known theoretically. In [34] it is shown that different reasonable choices give slightly different results, and none of them agree perfectly with a more fine scaled microscopic model. URDME and STEPS uses the same type of mesh, and the length parameter is chosen in the same way. URDME and STEPS does indeed give identical results for the period, up to the resolution of the experiment. Additional analysis can be found in [55, 56].

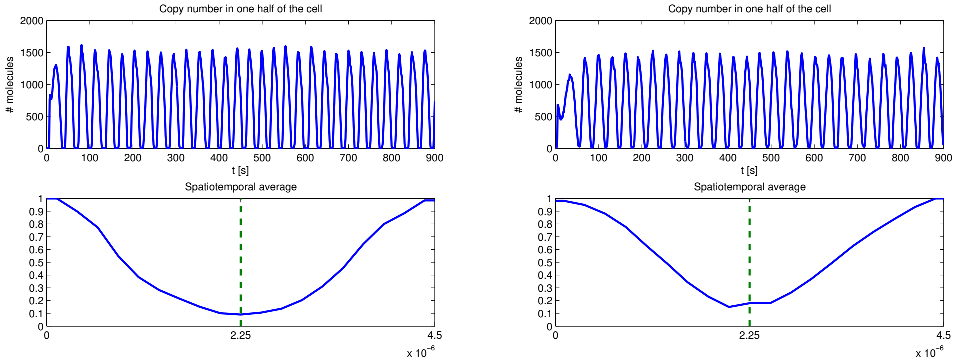

Figure 2: Temporal average profiles for simulation with URDME (left) and MesoRD (right), membrane diffusion constant $10^{-14}\ m^2/s$

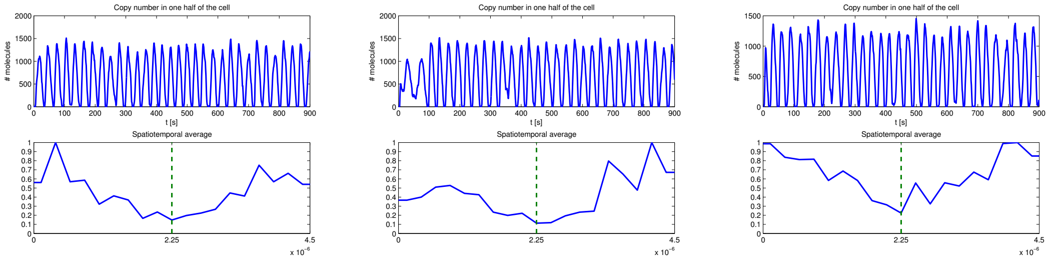

Figure 3: Oscillations and temporal average profiles for representative simulations with URDME (left), STEPS (center) and MesoRD (right) for the model with no membrane diffusion. The variation in the temporal average is much larger when there is no surface diffusion, which can be seen in the temporal average profiles. All software give similar periods and amplitudes.
